# Identification of DNA damage response-related genes as biomarkers for castration-resistant prostate cancer

**DOI:** 10.1038/s41598-023-46651-6

**Published:** 2023-11-10

**Authors:** Masashi Oshima, Ken-ichi Takayama, Yuta Yamada, Naoki Kimura, Haruki Kume, Tetsuya Fujimura, Satoshi Inoue

**Affiliations:** 1 Department of Systems Aging Science and Medicine, Tokyo Metropolitan Institute for Geriatrics and Gerontology, 35-2 Sakaecho Itabashi-ku, Tokyo, 173-0015 Japan; 2 Department of Urology, Jichi Medical University, Tochigi, Japan; 3 Department of Urology, Jichi Medical University Saitama Medical Center, Saitama, Japan; 4 Department of Urology, Graduate School of Medicine, The University of Tokyo, Tokyo, Japan; 5 Division of Systems Medicine and Gene Therapy, Saitama Medical University, Saitama, Japan

**Keywords:** Biochemistry, Cancer, Biomarkers, Pathogenesis

## Abstract

Although hormone therapy is effective for the treatment of prostate cancer (Pca), many patients develop a lethal type of Pca called castration-resistant prostate cancer (CRPC). Dysregulation of DNA damage response (DDR)-related genes leads to Pca progression. Here, we explored DDR-related signals upregulated in CRPC tissues. We analyzed the gene expression profiles in our RNA-sequence (RNA-seq) dataset containing benign prostate, primary Pca, and CRPC samples. We identified six DDR-related genes (Ribonuclease H2 Subunit A (*RNASEH2A*), replication factor C subunit 2 (*RFC2*), *RFC4*, DNA Ligase 1 (*LIG1*), DNA polymerase D1 (*POLD1*), and DNA polymerase E4 (*POLE4*)) that were upregulated in CRPC compared with Pca tissues. By analyzing public databases and validation studies, we focused on RFC2 as a new biomarker. Functional analysis demonstrated that silencing of RFC2 expression inhibited cell proliferation and induced the expression of DNA damage and apoptosis markers in CRPC model cells. Furthermore, immunohistochemical (IHC) analysis revealed that high expression of RFC2 protein correlated with poor prognosis in patients with Pca and increased expression in CRPC tissues compared with localized Pca. Thus, our study suggests that six DDR-related genes would be important for Pca progression. RFC2 could be a useful biomarker associated with poor outcomes of patients with Pca.

## Introduction

Prostate cancer (Pca) is one of the most frequently diagnosed cancers worldwide^1^. As most of Pca is androgen-dependent, androgen deprivation therapy (ADT) is performed for the advanced type of Pca^2, 3^. Although ADT is initially successful, some patients eventually present with biochemical and clinical evidence of resistance to treatment. This disease status of Pca is known as castration-resistant prostate cancer (CRPC)^4^. In CRPC, the androgen receptor (AR) is overexpressed or hyperactivated, resulting in activated downstream AR signals^5–8^. As CRPC is lethal^4^, several drugs, such as new types of anti-androgen drugs or taxane-based chemotherapy, have been developed to treat CRPC^9^. However, there is a need to develop new therapeutic agents because of their limited therapeutic effects^10^.

To identify common genetic alterations in CRPC, comprehensive analysis using exome sequencing revealed a high mutation frequency (22.7%) of DNA damage response (DDR)-related genes^11^. The DDR pathway involves multiple mechanisms that maintain genomic integrity against endogenous and exogenous DNA damage. The DDR pathway is important for cancer progression as dysregulation of the DDR pathway leads to genomic instability, increased mutation rates, and enhanced tumor heterogeneity^12–15^. Recently, a new drug targeting the DDR pathway, poly (adenosine diphosphate [ADP]-ribose) polymerase inhibitor (PARPi), was developed and demonstrated to be therapeutically useful against CRPC^16^. High frequencies of mutations have been observed in genes involved in homologous recombination repair (HRR), a DDR pathway, such as breast cancer1/2 (BRCA1/2) and ataxia telangiectasia mutated (ATM) in CRPC^17^. PARPi induces synthetic lethality by suppressing the function of PARPi, a key molecule in base excision repair (BER), which is an alternative DDR pathway for CRPC where HRR does not function properly due to specific genetic mutations^16, 18^. In addition, new anticancer therapies targeting various DDR pathways have been developed, such as DNA-dependent protein kinase catalytic subunit (DNA-PKcs) inhibitors against non-homologous end joining (NHEJ) and ataxia telangiectasia and Rad3-related (ATR) inhibitors against HRR^19^. Recent studies have revealed that high AR expression is strongly correlated with high DDR pathway-related gene expression^20^. High expression of DDR-related genes has been reported to be associated with Pca progression^20^, suggesting the importance of the DDR pathway in the pathogenesis of CRPCs with elevated AR activity and expression. This indicates that targeting DDR pathway could be a promising strategy for treating CRPC.

We previously used RNA-sequencing (RNA-seq) to understand the expression profiles in benign prostate, localized Pca, and CRPC tissues^21^. Here, in this study we investigated aberrantly expressed DDR-related signals during the development of CRPC and reveal its role in the progression of prostate cancer to identify new molecular signal associated with the development of castration-resistance. We then obtained six candidate DDR-related genes whose expression was upregulated in CRPC tissues compared to that in localized Pca. In particular, we focused on replication factor C subunit 2 (RFC2), which loads proliferating cell nuclear antigen (PCNA), the central molecule of DDR, into DNA and catalyzes nucleotide excision repair (NER), one of the DDR pathways. By analyzing its expression level, function, and clinical role in Pca progression, we demonstrated that RFC2 would be a useful biomarker associated with poor outcomes of patients with Pca.

## Results

### mRNA expressions of DDR-related genes are upregulated in CRPC compared to those in Pca

To identify a cluster of upregulated genes in CRPC, we previously conducted directional RNA-seq analysis using clinical samples obtained from localized Pca and CRPC patients^21^. We used RNA samples obtained from prostate cancer patients by radical prostatectomy, transurethral resection of the prostate, autopsy, and trans rectal biopsy. Benign prostate (Benign, N = 6), localized prostate cancer (Pca, N = 8), and castration-resistant prostate cancer (CRPC, N = 6) tissues were used for this RNA-seq analysis^21^. We then compared gene expression levels of CRPC tissues with localized Pca, demonstrating that comprehensive CRPC-specific alteration of signals in prostate cancer progression.

**Figure 1:**
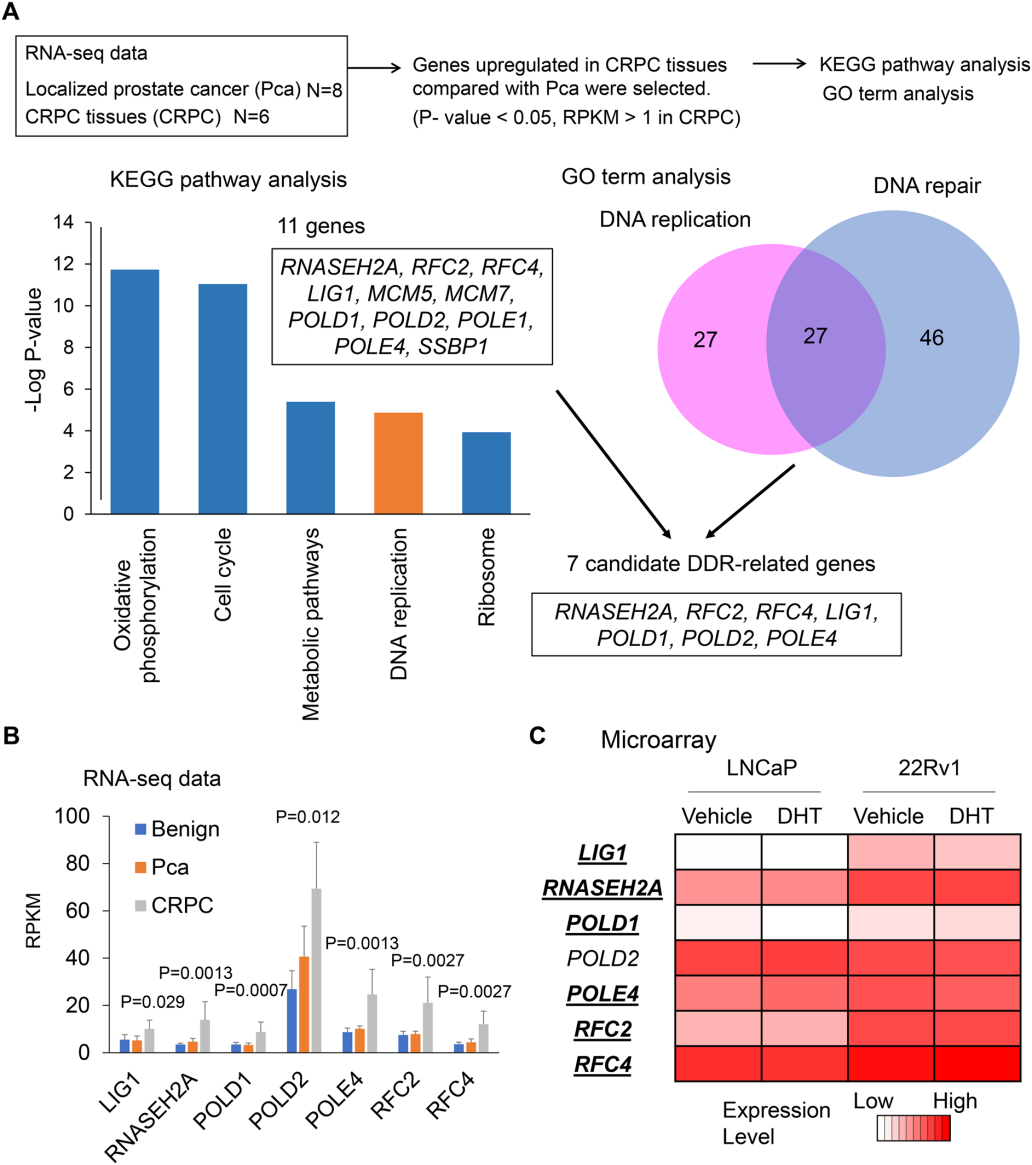
DNA damage response (DDR)-related genes are upregulated in castration resistant prostate cancer (CRPC). (**A**) Schematic depicting workflow of re-analysis of our RNA-seq data. Benign prostate (Benign, N = 6), localized prostate cancer (Pca, N = 8), and castration-resistant prostate cancer (CRPC, N = 6) tissues were used. Genes upregulated in CRPC tissues compared with localized prostate cancer (Pca) (RPKM > 1 in CRPC tissues, Mann–Whitney test, *P* < 0.05). Obtained protein-coding genes (919 gene symbols) were used for functional annotations in Kyoto Encyclopedia of Genes and Genomics (KEGG) pathway analysis and Gene Ontology (GO) term analysis. Seven overlapped genes associated with DDR (DNA repair, DNA replication) were selected. (**B**) mRNA expression levels of potential DDR-related genes in benign prostate, localized Pca and CRPC tissues. RNA sequence data^21^ was used to analyze the expression levels. The results are presented as mean and SD. *P*-value was determined by Mann–Whitney test between Pca and CRPC tissues. (**C**) mRNA expression levels of DDR-related genes in LNCaP and 22Rv1 cells. Data from microarray analysis in LNCaP and 22Rv1 cells^22^ is shown by the heat map. RPKM: reads per kilobase of exon per million mapped reads.

In the present study, a total of 919 protein-coding gene symbols were significantly upregulated (Mann–Whitney *U* test, *P* < 0.05, RPKM > 1) in CRPC tissues compared with Pca tissues. We then evaluated pathway enrichment in this group of genes using The Database for Annotation, Visualization, and Integrated Discovery (DAVID). Kyoto Encyclopedia of Genes and Genomes (KEGG) pathway analysis of these upregulated genes revealed significant enrichment of DNA replication-associated genes, such as RNASEH2A, RFC2, RFC4, LIG1, MCM5, MCM7, POLD1, POLD2, POLE1, POLE4, and SSBP1, suggesting that these DNA replication-associated genes have important roles in the development of CRPC (Fig. 1A). Similarly, Gene Ontology (GO) term analysis of these upregulated genes revealed that 27 genes were involved in both DNA replication and DNA repair. By combining both KEGG analysis and GO term analysis, seven genes (RNASEH2A, RFC2, RFC4, LIG1, POLD1, POLD2, and POLE4) were identified as new biomarker genes upregulated in CRPC compared to localized Pca and associated with DNA replication and DNA repair (Fig. 1B).

We then compared the expression levels of these seven genes in the 22Rv1 cells, an AR-positive CRPC model, and in LNCaP cells, a hormone therapy-sensitive model, using microarray data obtained in our previously conducted analysis^22^. We found that mRNA expression levels of six genes were upregulated in 22Rv1 cells compared to those in the LNCaP cells, indicating that these genes are upregulated in AR-positive CRPC compared with hormone therapy-sensitive Pca. Therefore, we assumed these genes as candidates for important DDR-related genes in CRPC development (Fig. 1C).

**Figure 2:**
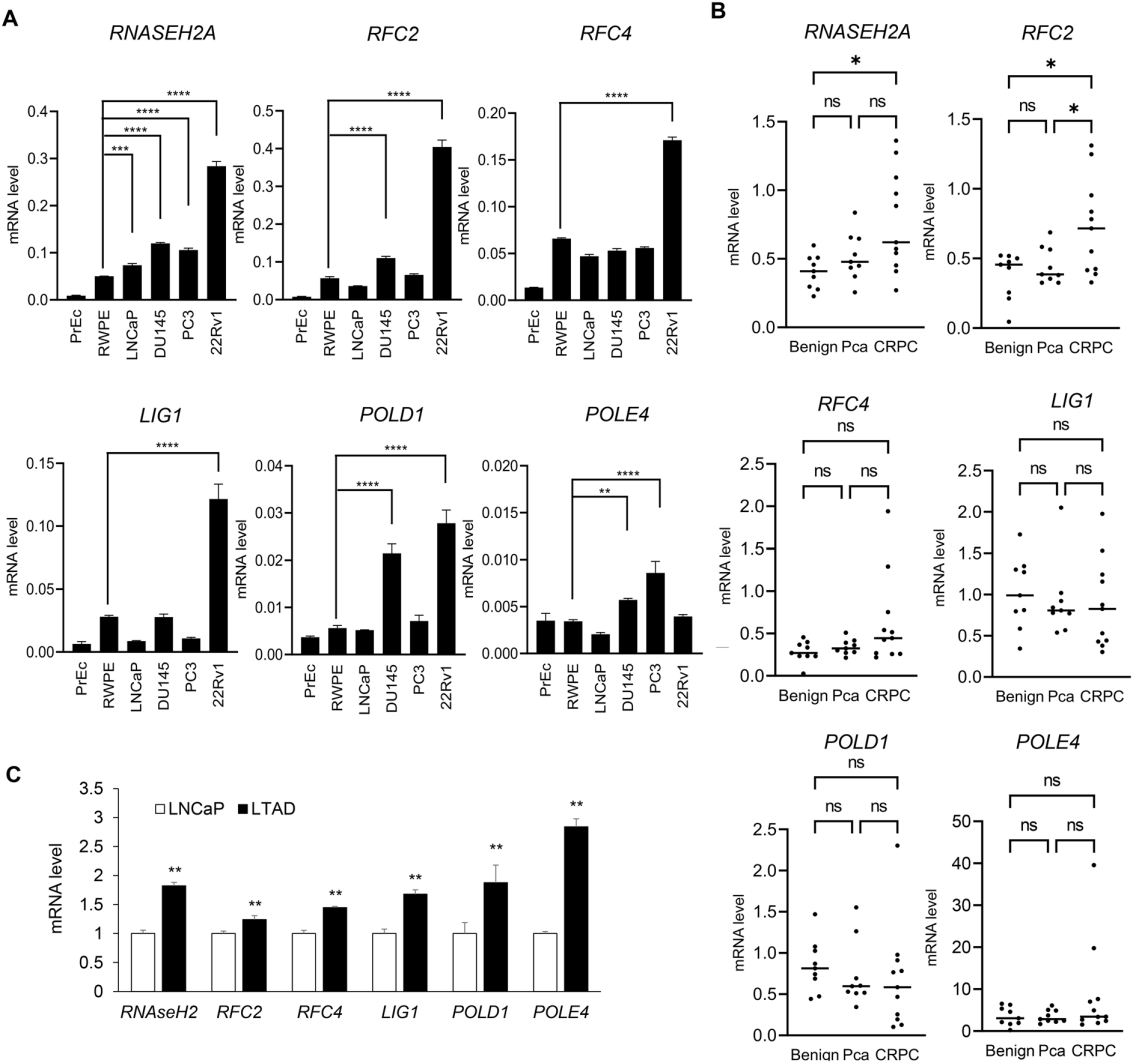
Screening assays for promising DDR-related genes upregulated in CRPC. (**A**) DDR-related gene expression levels in human prostate cell lines. Gene expression level (normalized to the housekeeping gene glyceraldehyde-3-phosphate dehydrogenase (GAPDH)) was assessed by quantitative real-time qRT-PCR in nonmalignant human prostate cell lines (PrEc, RWPE) and in human cancer prostate cell lines (LNCaP, DU145, PC3 and 22Rv1). The results are presented as mean and SD. One-way ANOVA and Dunnet’s post-doc tests were performed to obtain P-values. **P < 0.01, ***P < 0.001, ****P < 0.0001, compared with RWPE. (**B**) DDR-related gene expression levels in clinical prostate samples. RNA was extracted from each prostate clinical sample (Benign: N = 9, Pca: N = 9, CRPC: N = 11). We then measured mRNA expression levels of each DDR-related genes by qRT-PCR. The bars indicate the mean value. *P*-value was determined by Mann–Whitney test. *P < 0.05, ns; not significant. Pca: localized prostate cancer. (**C**) DDR-related gene expression levels in long term androgen deprivation (LTAD) and parental LNCaP cells. Gene expression level was assessed by quantitative real-time qRT-PCR. The results are presented as mean and SD (N = 3). Two-sided Student’s t-test was performed to obtain *P*-value. **P < 0.01, compared with LNCaP.

Next, we examined the expression of these DDR-related genes in human prostate cell lines. The mRNA expression levels were then analyzed by qRT-PCR using total RNA extracted from normal prostate cell lines (PrEc and RWPE) and Pca cell lines (LNCaP, DU145, PC3, and 22Rv1). We observed that the mRNA expression levels of RNASEH2A were significantly upregulated in Pca cell lines as compared to those in RWPE, and 22Rv1 cells exhibited the highest expression level of RNASEH2A among these cell lines. Similarly, we observed significantly upregulated mRNA expression of four genes (RFC2, RFC4, LIG1, and POLD1) in Pca cell lines compared to those in PrEc cells, and the highest expression levels were detected in 22Rv1 cells. Although POLE4 was also significantly upregulated in several cell lines, the highest expression level was detected in PC3 cells but not in 22Rv1 cells (Fig. 2A).

We further validated the results of RNA-seq analysis using another set of clinical samples. We measured the mRNA expression levels of potential DDR-related genes in benign prostate tissue (N = 9), localized Pca (N = 9), and CRPC (N = 11) using qRT-PCR. We observed that the expression levels of RNASEH2A (*P* = 0.011) and RFC2 (*P* = 0.011) were significantly increased in CRPC tissues compared with those in benign tissues. In particular, RFC2 was significantly upregulated in CRPC tissues compared to localized Pca tissues (*P* = 0.047) (Fig. 2B).

To determine the change of gene expression between hormone-sensitive Pca and CRPC, we used another CRPC model cells derived from LNCaP cells, long term androgen deprivation (LTAD) cells. We then compared the expression levels of DDR-related genes in LTAD cells with parental LNCaP. The result of qRT-PCR analysis revealed induced expression of these genes in LTAD cells compared with LNCaP (Fig. 2C), suggesting the important role of these genes in CRPC development.

### Expression and prognostic analysis of DDR-related genes in CRPC using public databases

The expression profile of these candidate genes in Pca and normal tissues adjacent to tumors was investigated using the online platform of Gene Expression database of Normal and Tumor Tissues 2 (GENT2) (http://gent2.appex.kr/gent2/) constructed from HG-U133A microarray data^23^. We noticed that only the expression level of RFC2 increased in Pca tissues compared with that in benign prostate tissues (Supplementary Fig. S1A). Next, differential expression levels of DDR-related genes were analyzed using three datasets (GSE35988, GSE6919, and GSE3325) downloaded from the Gene Expression Omnibus (GEO). The expression levels of DDR-related genes among the three groups of benign, Pca, and metastatic CRPC tissues were compared (Supplementary Fig. S1B–D). We found that only RNASEH2A was significantly upregulated in CRPC compared with Pca in all datasets. RFC2, RFC4, LIG1, and POLD1 were upregulated in two datasets, whereas POLE4 was not upregulated in any dataset (Supplementary Fig. S1E). Furthermore, gene expression profiling interactive analysis 2 (GEPIA2) (http://gepia2.cancer-pku.cn/) was used to evaluate the relationship between overall survival (OS) or biochemical failure-free survival (BFFS) and DDR-related gene expression for Pca in The Cancer Genome Atlas (TCGA) project^24^. We divided the patients into two groups based on the expression level of each DDR-related gene (cutoff-high: 25%, cutoff-low: 75%) and compared the prognosis of patients in the two groups. The high-expression group showed poor BFFS for RNASEH2A (*P* = 0.00013), RFC2 (*P* = 0.000021), RFC4 (*P* = 0.00094), LIG1 (*P* = 0.0011), and POLD1 (*P* = 0.00019) compared with the low expression group (Supplementary Fig. S2A). Moreover, we found that high RFC2 expression was associated with poor OS (*P* = 0.0056). In contrast, high POLE4 expression was not associated with poor OS (*P* = 0.53) or BFFS (*P* = 0.09) (Supplementary Fig. S2B).

### The role of DDR-related genes in CRPC cell proliferation and anti-apoptotic activity

**Figure 3:**
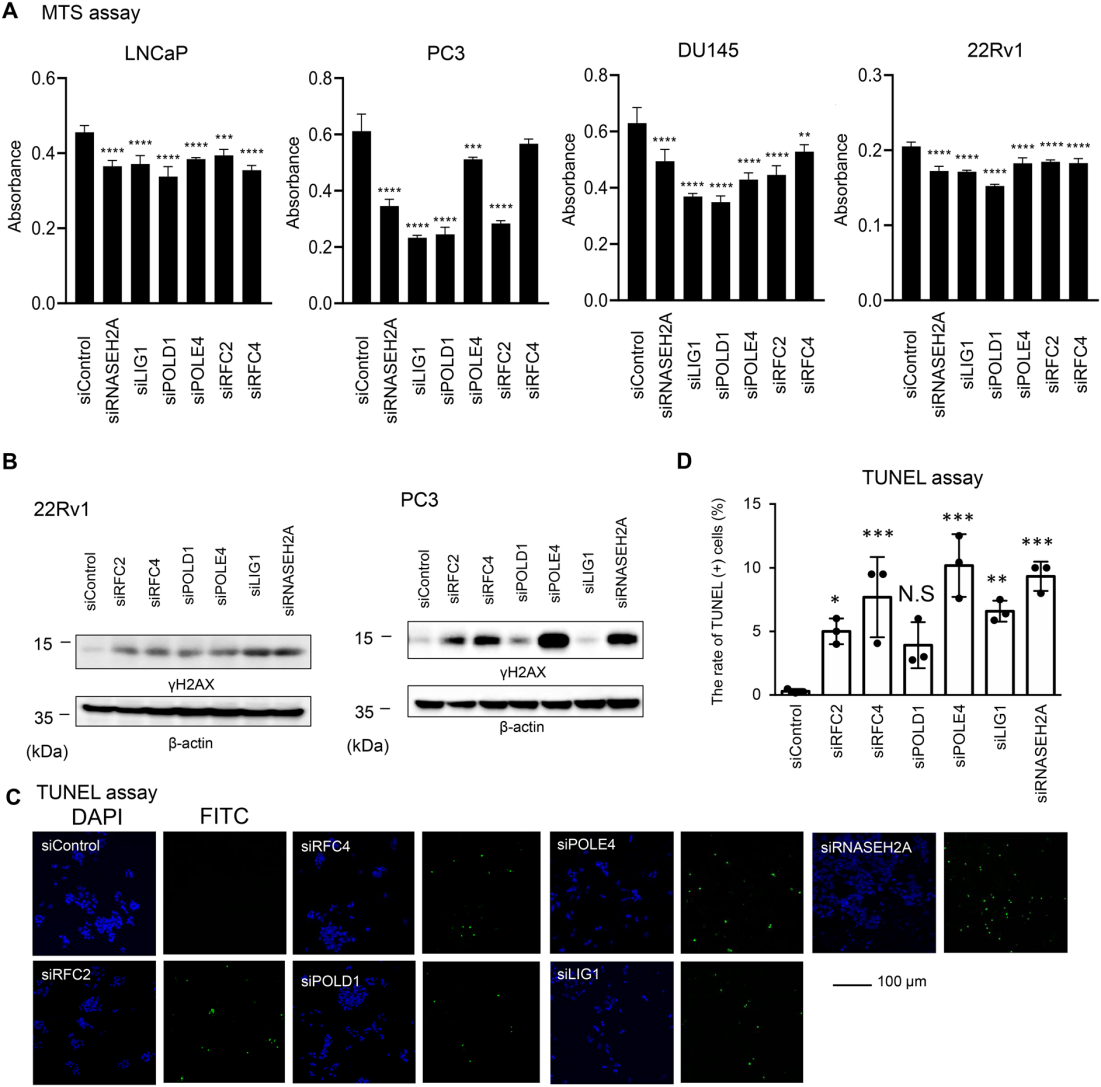
Knockdown of DDR-related genes results in accumulation of DNA damage, increased apoptosis and growth inhibition in CRPC cells. (**A**) Knockdown of DDR-related gene suppresses cell proliferation of Pca cell lines. After treated with 5 nM siControl or siDDR-related genes, MTS assay was carried out at day 3 (N = 4). The absorbance quantified in the plate reader is shown. The results are presented as mean and SD. One-way ANOVA and Dunnet’s post-doc tests were performed to obtain *P*-values. **P < 0.01, ***P < 0.001, ****P < 0.0001 compared with siControl. (**B**) Knockdown of DDR-related genes increases DNA damage in Pca cells. PC3 and 22Rv1 cells were transfected with 5 nM siControl or siDDR-related genes for 72 h. Western blot analysis for γH2AX was carried out and β-actin was used as a loading control. (**C**) TUNEL assay was performed to analyze the effect of siDDR-related genes in Pca cells. 22Rv1 cells were transfected with 5 nM siControl or siDDR-related genes for 72 h. Representative images of FITC positive cells treated with 5 nM siDDR-related genes or siControl for 72 h are showed. Bar = 100 μm. (**D**) Quantification of TUNEL positive cell (N = 3, biological replicates). One-way ANOVA and Dunnet’s post-doc tests were performed to obtain *P*-values (*P < 0.05, **P < 0.01, ***P < 0.001 vs siControl, *N.S* not significant). Data are presented as the mean ± SD.

To investigate the functional roles of these potential DDR-related genes in CRPC cell growth, an MTS assay was performed by treating LNCaP, PC3, DU145, and 22Rv1 cells with specific small interfering RNA (siRNA) to suppress the expression of each gene (siRNASEH2A, siRFC2, siRFC4, siLIG1, siPOLD1, and siPOLE4). We confirmed a substantial reduction in endogenous expression in PC3 cells treated with each siRNA targeting a DDR-related gene compared with the siControl by qRT-PCR analysis (Supplementary Fig. S3A). We observed that cell proliferation was significantly repressed in all cell lines treated with siRNASEH2A, siRFC2, siLIG1, siPOLD1, and siPOLE4 compared to that in siControl. Treatment with siRFC4 did not inhibit the growth of PC3 cells but inhibited the growth of LNCaP, DU145, and 22Rv1 cells (Fig. 3A).

To analyze the DDR pathway induced by these genes in Pca cells, we analyzed the expression of phosphorylated histone H2AX (γH2AX), known marker for DNA damage, in particular double strand breaks. Intriguingly, increase of γH2AX signal was observed in 22Rv1 and PC3 cells treated with siRNAs against DDR-associated genes excepting LIG1 in PC3 cells (Fig. 3B). Furthermore, we evaluated the effects of siRNA-mediated knockdown of DDR-associated genes on the induction of apoptosis by TUNEL assay. We then observed that knockdown of these genes excepting POLD1 significantly induced apoptosis in 22Rv1 cells (Fig. 3C,D). Thus, these results suggest that DDR-related genes upregulated in CRPC tissues have a role in preventing DNA damages and apoptosis.

### The role of RFC2 on proliferation of CRPC cells

**Figure 4:**
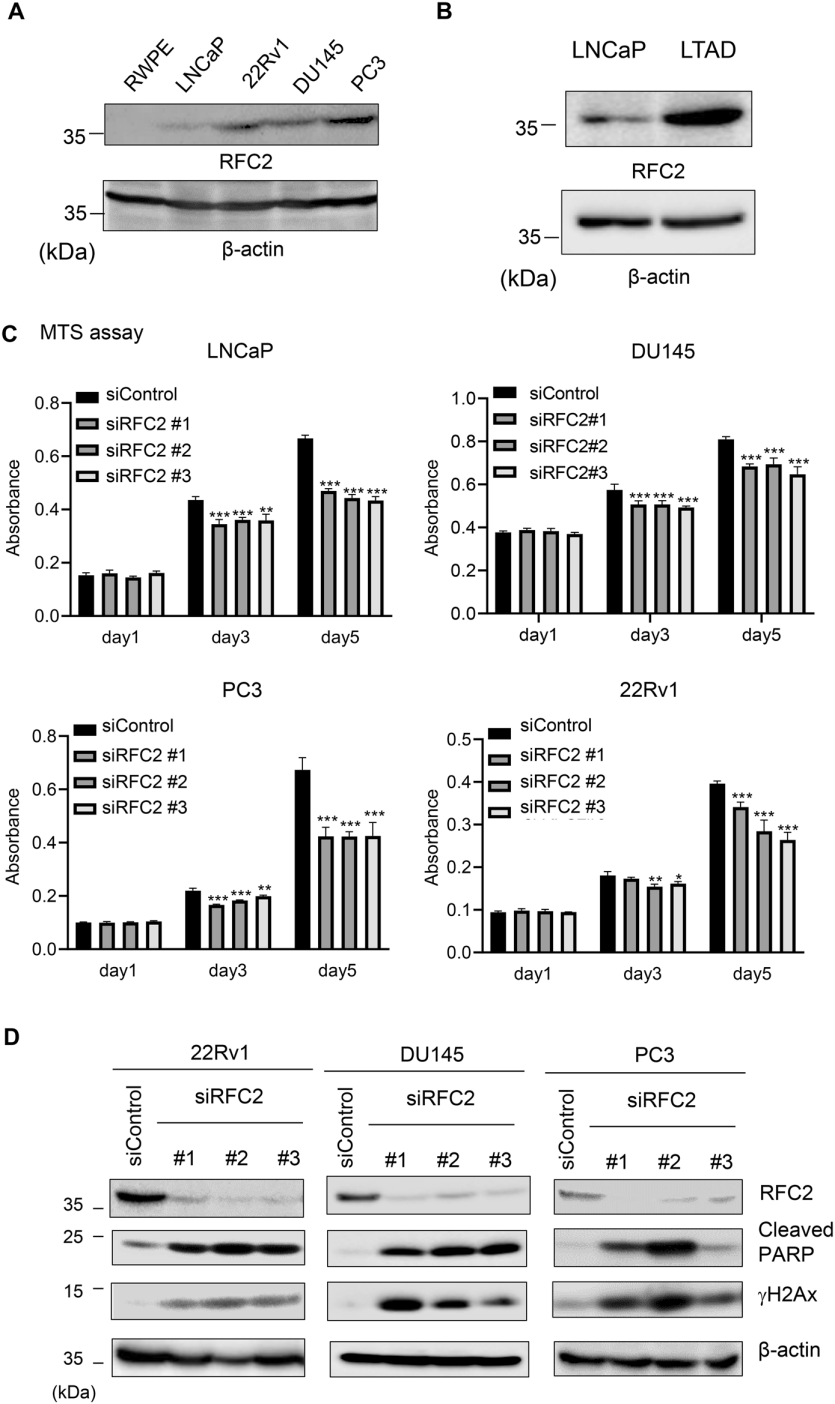
Knockdown of RFC2 results in accumulation of DNA damage, increased apoptosis and growth inhibition in CRPC cells. (**A**) The expression level of RFC2 at the protein level in multiple prostate cell lines. Lysates from five prostate cell lines (RWPE, LNCaP, 22Rv1, DU145 and PC3 cells) were used for Western blot analysis with specific antibodies to replication factor C subunit2 (RFC2). β-Actin was used as a loading control. (**B**) The expression level of RFC2 at the protein level in LTAD and parental LNCaP cells. Lysates were used for Western blot analysis with specific antibodies to replication factor C subunit2 (RFC2). β-Actin was used as a loading control. (**C**) Knockdown of RFC2 suppresses cell proliferation of Pca cell lines. LNCaP, DU145, PC3 and 22Rv1 cells were treated with 5 nM siControl or siRFC2 (#1, #2, and #3). MTS assay was carried out at the indicated time points (N = 6). The absorbance quantified in the plate reader is shown. The results are presented as mean and SD. One-way ANOVA and Dunnet’s post-doc tests were performed to obtain P-values. *P < 0.05, **P < 0.01, ***P < 0.001, compared with siControl. (**D**) Knockdown of RFC2 increases DNA damage and apoptosis in Pca cell lines. PC3, DU145 and 22Rv1 cells were transfected with 5 nM siControl or siRFC2 (#1, #2, and #3) for 48 h. Western blot analysis for RFC2, cleaved PARP and γH2AX was carried out and β-actin was used as a loading control.

Among the six DDR-related genes whose expression was upregulated in CRPC tissues, we recently reported that RNASEH2A overexpression maintains genomic stability to prevent R-loop-mediated apoptosis induction during Pca progression^22^. We then focused on RFC2 as the most promising DDR-related biomarker because mRNA expression level of RFC2 is upregulated in CRPC tissue compared with Pca, as validated by qRT-PCR analysis. It is also notable that mRNA expression of RFC2 is a poor prognostic factor for both BFFS and OS in a public database. We further evaluated the protein expression level of RFC2 in five prostate cell lines (RWPE, LNCaP, 22RV1, DU145, and PC3). Western blotting demonstrated that RFC2 was highly expressed in CRPC model cells (22RV1, DU145, and PC3) compared with hormone-sensitive Pca cells (LNCaP), in line with the high mRNA expression levels in CRPC tissues (Fig. 4A, Supplementary Fig. S4A). Moreover, we observed RFC2 protein level is elevated in LTAD cells compared with LNCaP cells (Fig. 4B).

Next, to analyze the functional roles of RFC2 in Pca cells, we evaluated the knockdown efficacy of multiple siRNAs targeting RFC2. We confirmed a substantial reduction in endogenous expression in PC3 and 22Rv1 cells treated with siRFC2 compared with siControl at mRNA level (Supplementary Fig. S3B). We evaluated the effect of RFC2 knockdown on Pca and CRPC cell growth. MTS assays were performed using LNCaP, PC3, DU145, and 22Rv1 cells transfected with siRFC2. We observed that cell growth was significantly repressed in all cell lines treated with the three sequences of siRFC2 compared with siControl (Fig. 4C). We then evaluated changes in DNA damage repair and apoptosis following knockdown of RFC2. The protein expression levels of RFC2, cleaved PARP and γH2AX in Pca cells treated with siControl or siRFC2 were also evaluated. Western blotting confirmed decreased expression of RFC2 at protein level by siRFC2 treatment. Of note, increased expression of cleaved PARP and γH2AX in DU145, PC3 and 22Rv1 cells were observed after RFC2 knockdown (Fig. 4D, Supplementary Fig. S4A). Similar result was also observed in another AR-overexpressing CRPC cell line, VCaP cells (Supplementary Fig. S4B,C).

### RFC2 is overexpressed in Pca, particularly in CRPC, and associated with poor prognosis

**Figure 5:**
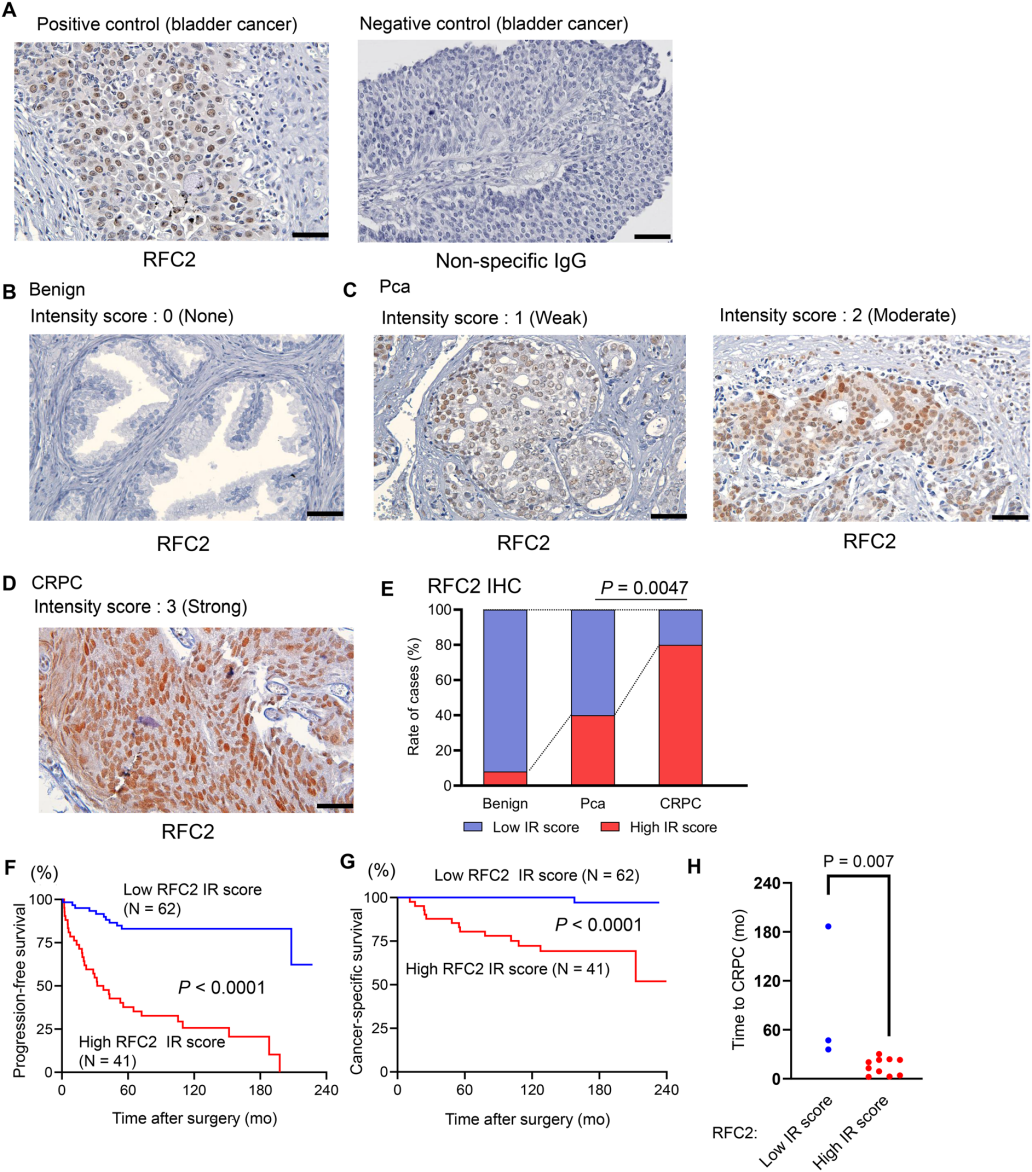
Immunohistochemical (IHC) analysis of RFC2 protein expression in Pca tissues. (**A**–**D**) Representative IHC images of RFC2 in Pca tissues. (**A**) Positive (anti-RFC2 antibody) and negative control (non-specific rabbit IgG antibody) using bladder cancer specimens. (**B**) Anti-RFC2 in benign prostate, intensity score 0. (**C**) Anti-RFC2 in Pca, intensity score 1 and 2. (**D**) Anti-RFC2 in CRPC, intensity score 3. Scale bar = 50 μm. (**E**) Rate of cases in which positive IR was detected by RFC2 IHC in benign (N = 12), Pca (N = 103), and CRPC (N = 15) tissues. Chi-squared test was done to calculate *P*-value. IR scores of 0‐4 and 5–8 were defined as low and high IR, respectively. (**F**,**G**) High immunoreactivity (IR) score of RFC2 is associated with poor prognosis of prostate cancer patients. Progression-free survival (**F**) and cancer-specific survival (**G**) of prostate cancer patients are shown (N = 103). Survival curve was obtained by Kaplan–Meier method and *P*-value was determined by log-rank (Mantel–Cox) test. (**H**) Evaluation of the time to CRPC diagnosis in CRPC cases (N = 15). The median follow-up time from radical prostatectomy to CRPC diagnosis was summarized in CRPC cases with low and high RFC2 IR. Mann–Whitney test was done to calculate *P*-value.

To further explore the clinical significance of RFC2 expression in Pca progression, we performed IHC analysis using tumor tissues obtained from 103 patients with localized Pca patients who had undergone radical prostatectomy. Representative immunostaining images of the RFC2 protein and controls are shown (Fig. 5A–D). RFC2 staining was also observed in nuclei. We then investigated the association between clinical parameters and RFC2 expression level. High RFC2 immunoreactivity (IR) (≥ 4) was significantly associated with higher pathological Gleason score (pGS), pathological T (pT) stage, and pathological N (pN) stage (*P* < 0.0001, *P* < 0.0001, and *P* = 0.0164, respectively, as shown in Table 1), although PSA was not significantly correlated with RFC2 expression. We also conducted IHC analysis using additional specimens of prostate tissue obtained from 12 benign prostate hyperplasia (benign) cases and 15 CRPC cases by prostate biopsy or transurethral resection of the prostate. We observed high RFC2 IR in 1 of 12 patients (8.3%) in benign tissues, 41 of 103 (39.8%) patients in Pca tissues, and 12 of 15 patients (80.0%) in CRPC tissues. The rate of high RFC2 IR was higher in CRPC tissues than in Pca (*P* = 0.0047, Fig. 5E). Taken together, these results demonstrated that RFC2 is overexpressed in advanced Pca at the protein level. High RFC2 IR was significantly associated with poor progression-free survival (*P* < 0.0001) and cancer-specific survival (*P* < 0.0001) of patients (Fig. 5F,G). These results were consistent with those of the prognostic analysis using public databases (Supplementary Fig. S2).

**Table 1:** Relationship between replication factor C subunit 2 (RFC2) immunoreactivity and clinicopathological findings in human prostate cancer patients (n = 103).

	RFC2 IR score (N = 103)		
	Low IR < 4 (N = 62)	High IR ≥ 4 (N = 41)	*P*-value
Age (y)	67.1 ± 5.9	66.0 ± 6.3	0.6904
Serum PSA level			
PSA < 10 ng/mL	33	20	0.8399
PSA ≥ 10 ng/mL	29	20	
Pathological Gleason score (pGS)			
pGS < 8	48	13	**< 0.0001**
pGS ≥ 8	14	28	
Pathological T stage			
pT < 3b	53	15	**< 0.0001**
pT ≥ 3b	9	26	
Pathological N stage			
pN = 0	58	31	**0.0164**
pN = 1	4	10	

Immunoreactivity (IR) score (0, 2–8) was obtained as the sum of the intensity and the area of IR. Intensity (0, none; 1, weak; 2, moderate; and 3, strong), Proportion (0, none; 1, < 1/100; 2, 1/100 to 1/10; 3, 1/10 to 1/3; 4, 1/3 to 2/3; and 5, > 2/3). Fisher’s exact test and Mann–Whitney *U* test were performed to determine *P*-value. *P*-value < 0.05 was considered to be statistically significant. IR scores of 0‐4 and 5–8 were defined as low and high IR, respectively. *PSA* prostate specific antigen. Note that serum PSA was not measured in one patient. Significant values are in bold.

In our IHC study, univariate analysis using the Cox proportional hazard model demonstrated that pGS, pT stage, pN stage, and RFC2 IR score were significant prognostic factors for cancer-specific survival (Table 2). In analyses using TCGA data including mRNA level expression level of RFC2 in Pca tissues, we obtained consistent results in the univariate analysis although RFC2 high expression was not independent prognostic factor for progression-free survival in multivariate study (Supplementary Table S1). However, multivariate analysis of these factors in our IHC study showed that the RFC2 IR score was the only independent prognostic factor (*P* = 0.047, Table 2). In addition, we investigated the time to CRPC in CRPC cases treated with radical prostatectomy and then found that RFC2 expression level is significantly associated with the time to CRPC of these cases (Fig. 5H). Therefore, these results raised the possibility that RFC2 overexpression is involved in prostate cancer progression and survival of patients.

**Table 2:** Univariate and multivariate analyses for cancer-specific survival in prostate cancer patients.

Parameters	Univariate		Multivariate	
	HR (95% CI)	*P*-value	HR (95% CI)	*P*-value
PSA (≥ 10 ng/mL vs < 10 ng/mL)	0.61 (0.20–1.82)	0.3759		
Pathological Gleason score (pGS) (pGS ≥ 8 vs pGS < 8)	21.23 (2.77–162.65)	**0.0033**	5.36 (0.52–55.19)	0.1578
T stage (T ≥ 3b vs < 3b)	13.49 (3.01–60.46)	**0.0007**	2.05 (0.33–12.77)	0.4415
N stage (N = N1 vs N0)	5.49 (1.90–15.91)	**0.0017**	1.58 (0.51–4.86)	0.4267
RFC2 IR score (IR ≥ 4 vs < 4)	23.19 (3.03–177.40)	**0.0025**	8.70 (1.03–73.63)	**0.0470**

Univariate and multivariate analyses were evaluated using Cox’s proportional hazard model.* P*-value < 0.05 was considered to be statistically significant. Multivariate analysis showed that RFC2 IR score was significantly associated with cancer-specific survival. *CI* confidence interval, *IR score* immunoreactivity score, *HR* hazards ratio, *PSA* prostate specific antigen, *RFC2* replication factor C subunit 2. Significant values are in bold.

## Discussion

In this study, we demonstrated that multiple DDR-related genes involved in DNA repair and DNA replication are highly upregulated in CRPC tissues compared to localized Pca. We re-analyzed our RNA-seq data using clinical samples. While the number of CRPC samples was limited, a subset of protein-coding genes was determined to be significantly upregulated in CRPC tissues compared with Pca tissues, showing comprehensive CRPC-specific alteration of signals. Six candidate genes (RNASEH2A, RFC2, RFC4, LIG1, POLD1, and POLE4) were obtained as potential DDR-related biomarkers of CRPC development. Moreover, by knocking down the expression of these genes, we showed that they regulate CRPC cell growth and apoptosis. By combining several screening analyses, we focused on RFC2 as a potential biomarker. To the best of our knowledge, this is the first study to examine the function and prognostic values of RFC2 in Pca progression. Previous studies reported higher expression of RFC2 in various tumor tissues, such as glioblastoma^25^, hepatocellular carcinoma^26^, nasopharyngeal carcinoma^27^, and colorectal cancer^28^, than in normal tissues. Recent analysis of publicly available gene expression database (microarray and RNA-seq) reported that RFC2 was generally highly expressed in many types of cancer tissues compared with normal tissues, including glioma and Pca^29^ Our results demonstrated that RFC2 is overexpressed in Pca, particularly in CRPC, at both the mRNA and protein levels, which is consistent with the results of previous studies. Thus, our findings indicate that the upregulation of RFC2 could be a useful biomarker of Pca progression.

Notably, IHC analysis showed that high expression of RFC2 was correlated with poor prognosis of Pca patients. We found some limitations in our survival analysis. This survival analysis used cancer-specific survival of patients in our cohort. Because our cohort represents aggressive form of disease, cancer-specific survival could be analyzed. It is reported that prostate cancer in Asia, and particularly in Japan, historically presented at a more advanced stage compared to Western countries, largely due to the limited spread of PSA screening during earlier years^30^. Our cohort, comprising cases that underwent total prostatectomy between 1987 and 2001, reflects this trend. We found 70 Pca cases with T3–T4 of pathological T stage among 103 cases, highlighting a high proportion of advanced cancers. We therefore speculate that this accounts for the high PSA recurrence rate (44 out of 103 cases) and the 14 instances of prostate cancer-related mortality in our study. Another limitation is that multivariate study by using TCGA data including gene expression profile at mRNA level in Pca tissues suggested that high expression of RFC2 mRNA was not independent prognostic factor for progression-free survival. However, our IHC study indicated that the RFC2 IR score was the only independent prognostic factor. This result might arise because the TCGA data included mRNA expression profiles. In contrast, our IHC study measured RFC2 expression levels at the protein level. Thus, future RFC2 IHC study using other cohorts, including those from other countries, would be helpful for understanding the importance of RFC2 in pathogenesis of advanced prostate cancer.

We further explored the role and function of RFC2 in Pca progression. Although we validated that the expression level of RFC2 was increased significantly in CRPC tissues compared with Pca in our qRT-PCR analysis, the difference was only 1.87-fold. However, significant upregulated expression in RNA-seq data as well as IHC study for RFC2 was also observed. Our cell growth assay and clinical data suggested that RFC2 is necessary for prostate cancer cell growth. The present results showed a higher expression level of RFC2 in several CRPC model cells such as 22Rv1, DU145 and PC3 cells than in hormone-sensitive LNCaP cells. In addition, we evaluated the expression level of RFC2 in androgen-starved condition by using LTAD cells and its parental LNCaP cells. RFC2 protein level is upregulated in LTAD compared with parental LNCaP cells. We revealed that knockdown of RFC2 accumulated DNA damage, and induced apoptosis markers in CRPC model cells. Therefore, we estimated that RFC2 has an important role in CRPC cells. This notion was also supported by our clinical results, which showed that the expression of RFC2 protein increased during disease progression. Progression-free and cancer-specific survivals in our cohort were assessed in patients who received prostatectomy, not androgen-deprivation therapy. Notably, we found the shorter time to CRPC diagnosis is significantly associated with high RFC2 expression level in CRPC cases. Taken together, our findings indicated that increased RFC2 expression is involved in acquiring resistance to ADT in Pca progression to CRPC.

We identified six DDR-related genes as putative biomarkers for predicting poor outcomes in patients with Pca. Mechanistically, these genes are essential for DNA repair, thereby maintaining the genomic integrity. Among them, RNASEH2A is a subunit of RNase H2, a major isoform of RNase H, which degrade DNA:RNA hybrid that deletes RNA mishybridization^22^. LIG1 is an ATP-dependent DNA ligase involved in DNA replication, recombination, and the base excision repair process by joining interruptions in the phosphodiester backbone of DNA^31^. As previously reported, LIG1 is highly expressed in various types of cancers for rapid proliferation of cancer cells^31^. RFC is a DNA-binding protein responsible for DNA replication, DNA repair such as mismatch repair (MMR) and NER, and regulation of cell growth and cell cycle checkpoints^32^. RFC acts as a clamp loader that loads PCNA onto a primer-bound DNA template to form a DNA–RFC–PCNA–DNA polymerase complex^33, 34^. PCNA is a homotrimeric protein, and each has a protein-binding site, forming a replication/repair fork (RF) that anchors proteins involved in DDR. RFC is a five-subunit complex comprising one large subunit RFC1 (140 kDa) and four small subunits (RFC2 (40 kDa), RFC3 (38 kDa), RFC4 (37 kDa), and RFC5 (36 kDa))^35^. Each RFC subunit is biologically activated in various cancers and plays important roles in cancer cell growth, progression, invasion, and metastasis^32^. Although it is expected to be a prognostic factor in cancer, the prognostic value will depend on the cytological and histological characteristics of the tumor and whether each RFC subunit functions as an oncogene or tumor suppressor gene^32^.

In CRPC, DDR-related gene mutations are common, suggesting the potential for personalized therapeutic strategies targeting the DDR pathway^11^. Although PARPi has been developed as a treatment option for CRPC targeting DDR, which exerts clinical benefits, resistance to PARPi has also been reported^36^. Several mechanisms of PARPi resistance have been reported, including RF stabilization^37^. When PARPi acts on BRCA-mutated cancer cells, it causes synthetic lethality through inhibition of the DDR pathway, while simultaneously inhibiting meiotic recombination 11 (MRE11), which degrades RF by blocking its interaction. Thus, RF is stabilized, facilitating cell survival by relieving replication stress^38–41^. As RFC2 acts as a cell cycle checkpoint and stabilizes RF, we expect that RFC2 inhibition could be a potential therapeutic strategy for CRPC resistant to PARPi. Furthermore, as the DNA single-strand break repair pathway is essential for cell survival as an alternative pathway in cancer cells with BRCA mutations, inhibition of the NER pathway by RFC2 in addition to PARPi may have additional therapeutic efficacy for CRPC treatment. Similarly, DDR-related genes we identified may serve as a promising therapeutic target in CRPC. These DDR-related genes function at different stages in the DDR process. Specifically, we expect that inhibition of the DDR pathway tailored to the individual mutation profile, and in combination with irradiation, radioligands, PARP inhibitors, and DNA-damaging anticancer drugs such as cisplatin may enhance the sensitivity of existing therapeutic agents. It would be interesting to investigate how inhibition of DDR genes is effective for the treatment of advanced Pca in the future study.

In conclusion, we identified six DDR-related genes which are associated with the treatment-resistance of Pca and the progression of Pca to CRPC. Among them, RFC2 expression is necessary for the growth of CRPC cells and has a preventive effect on the apoptosis and DNA-damage. Overexpression of RFC2 could serve as a biomarker correlated with Pca progression.

## Materials and methods

### Clinical samples

To perform quantitative reverse transcription polymerase chain reaction (qRT-PCR) analysis, we obtained human prostate samples (benign (N = 9), localized Pca (N = 9), and CRPC (N = 11)) from surgeries and biopsies conducted at the Jichi Medical University (Tochigi, Japan) and the University of Tokyo (Tokyo, Japan). For immunohistochemical (IHC) analysis, human formalin-fixed paraffin-embedded benign prostate hyperplasia (N = 16), localized Pca (N = 103), and CRPC (N = 21) tissues were collected from Jichi Medical University and the University of Tokyo. Several clinical parameters of patients including PSA are summarized in Table 1. This study was approved by the institutional ethical committee of Jichi Medical University (No. 18-15) and the University of Tokyo (No. G10044). This study was conducted in accordance with the Declaration of Helsinki. Written informed consent was obtained from all patients before enrollment.

### Cell culture and reagents

PrEC cells were obtained from Lonza Bioscience (Basel, Switzerland) and cultured according to the manufacturer’s instruction. Other prostate cell lines were obtained from American Type Culture Collection (ATCC, Manassas, VA, USA). Short tandem repeat (STR) analysis was performed to authenticate the cell lines used in the present study. Also, these cells have been checked for Mycoplasma contamination using real-time PCR performed by Funakoshi Co., Ltd. (Tokyo, Japan).All cell lines were cultured at 37 °C in a humidified air and 5% CO_2_ atmosphere. Also, we routinely checked for mycoplasma contamination using a PCR-based kit (Mycoplasma Detection Kit, Jena Bioscience, Jena, Germany). RWPE, LNCaP, PC3, DU145 and 22Rv1 cells were cultured in RPMI medium (Nacalai Tesque, Kyoto, Japan). VCaP cells were cultured in DMEM medium (Nacalai Tesque). All mediums were supplemented with 10% fetal bovine serum (FBS), 50 U/mL penicillin, and 50 μg/mL streptomycin. LTAD cells were established as described^21^ and maintained in phenol red free RPMI medium supplemented with 10% charcoal–dextran stripped FBS, 50 U/mL penicillin, and 50 μg/mL streptomycin.

### RNA-seq data and bioinformatics analysis

We used our RNA-seq data including benign prostate (Benign, N = 6), localized prostate cancer (Pca, N = 8), and castration-resistant prostate cancer (CRPC, N = 6) tissues^21^. This RNA-seq data has been deposited in the Japanese Genotype–phenotype Archive (JGA) under accession code JGAS00000000198. The expression levels of the mapped transcripts were normalized to reads per kilobase of exon per million mapped reads (RPKM) to facilitate comparison among different samples. We explored genes highly expressed in CRPC samples by performing Mann–Whitney *U* test and identified significantly upregulated genes (protein-coding gene symbols) in CRPC samples compared with Pca (*P* < 0.05, RPKM > 1 in CRPC tissues). Differentially expressed genes were subjected to Gene Oncology (GO) and Kyoto Encyclopedia of Genes and Genomes (KEGG) pathway analyses using the DAVID software (https://david.ncifcrf.gov/).

We also used publicly available RNA-seq and microarray data for validation study. The online platform of Gene Expression database of Normal and Tumor Tissues 2 (GENT2) (http://gent2.appex.kr/gent2/)^23^ was used to compare the expression profile of candidate genes in Pca with normal tissues adjacent to tumors. Differential expression levels of DDR-related genes were analyzed using three datasets (GSE35988, GSE6919, and GSE3325) downloaded from the Gene Expression Omnibus (GEO). The *P* value was determined by Mann–Whitney *U* test. Gene expression profiling interactive analysis 2 (GEPIA2) (http://gepia2.cancer-pku.cn/) was used to evaluate the relationship between overall survival (OS) or biochemical failure-free survival (BFFS) and DDR-related gene expression for Pca in The Cancer Genome Atlas (TCGA) project^24^. We divided the patients into low (Bottom 75% cutoff) and high expression groups (Top 25% cutoff). For univariate and multivariate analysis of Pca patients using TCGA data, we downloaded clinical information and RFC2 expression profile of Pca samples (N = 491) from cBioPortal database (https://www.cbioportal.org).

### Small interfering RNA (siRNA) transfection

We transfected cells with control siRNA or siRNAs (5 nM) targeting DDR-related genes. The transfection reagents Lipofectamine RNAi MAX (Thermo Fisher Scientific, Waltham, MA, USA) was used according to the manufacture's protocol and cells were incubated 48–72 h post transfection. We purchased control siRNA and siRNAs (siRFC2#1: s11944, siRFC2#2: s11945, siRFC2#3: s11946, siRFC4: s11952, siLIG1: s8173, siPOLD1: s616, siPOLE4: s32216) from Thermo Fisher Scientific. Designed sequences of siRNASEH2 were described before^22^.

### Cell proliferation assay

LNCaP, PC3, DU145 and 22Rv1 cells were seeded in 96-well plates (1000 cells/well) and transfected with siRNAs after 24 h incubation. 10 µL of CellTiter 96 aqueous one solution cell proliferation assay (MTS) solution (Promega, Madison, WI, USA) was added to cells and incubated for 90 min. The 490-nm absorbance was then quantified in the plate reader. Assays were carried out in quadruplicate, and data of measured absorbance are presented as mean and SD.

### RNA extraction and quantitative reverse transcription polymerase chain reaction (qRT-PCR)

Total RNA was isolated and complementary DNA (cDNA) was synthesized as described^21^. We performed laser microdissection to obtain benign and cancer cells from clinical samples as described^21, 42^. Amplification was performed using Applied Biosystems Step one plus real-time PCR System (Thermo Fisher Scientific) based on KAPA SYBR Green (KAPA Biosystems, Boston, MA, USA). Fold changes for experimental groups relative to the loading control (GAPDH) were calculated by ΔΔCt method.

The primer sequences were described before23 or designed as followed:

- RFC2 forward: 5′-TCCGGTACACAAAGCTGACC-3′
- RFC2 reverse: 5′-GGCTCGTCACAGACCTTGAA-3′
- RFC4 forward: 5′-AAGTCGCTCAGATGGGAAGC-3′
- RFC4 reverse: 5′-TCTGACAGAGGCTTGAAGCG-3′
- LIG1 forward: 5′-GGAATGGAGTGGTGTCCGAG-3′
- LIG1 reverse: 5′-TGGGAGAGGTGTCAGAGAGG-3′
- POLD1 forward: 5′-ATCCGGTTCATGGTGGACAC-3′
- POLD1 reverse: 5′-GGCTCAGGGAAGATGCCTTT-3′
- POLE4 forward: 5′-GAGGGACCTGCTGGGGAG-3′
- POLE4 reverse: 5′-AGCGCAACAGTAGGCATCTT-3′

### Western blot analysis

Whole-cell lysates were prepared using lysis buffer (50 mM Tris–HCl [pH 8.0], 150 mM NaCl, 1% NP‐40, protease inhibitor cocktail (Nacalai Tesque, Kyoto, Japan). Western blot analysis was performed as described^21^. The following antibodies were used in this study: anti-RFC2 antibody (ab251796, Abcam, Cambridge, UK), anti-cleaved PARP antibody (ab32064, Abcam), anti-gamma H2AX antibody (ab11174, Abcam), anti-β-actin antibody (Wako, Tokyo, Japan). After reaction with primary antibodies at 4 °C overnight, membranes were incubated with HRP-conjugated anti-rabbit or anti-mouse IgG secondary antibodies for 1 h at room temperature. The membranes then were reacted with Pierce ECL Western Blotting Substrate (ECL Solution Kit; Thermo Fisher Scientific). Band signals were detected by using Fusion Solo S System (Vilber-Lourmat, Osaka, Japan), and the results were quantified using Image J software.

### TdT-mediated UTP nick-end labeling (TUNEL) assay

The DEADEND Fuorometric TUNEL system (Promega, Madison, WI, USA) was used to evaluate apoptosis. Cells (3 × 10^4^) were seeded on a cover glass in 24-wells plates 24 h before the treatment with siRNAs. After 72 h of treatment, the cells were stained with TUNEL according to the manufacturer’s protocol. The 4′,6-diamino-2-phenylindole (DAPI) (Nakarai, Tokyo, Japan) was used to stain nuclei. We observed TUNEL-positive cells using confocal laser scanning microscopy (Fluoview FV10I, OLYMPUS, Tokyo, Japan) and counted the number of cells in three fields. Data were evaluated by calculating the mean ± SD.

### Immunostaining and immunohistochemical assessment

Immunohistochemistry analyses were performed on formalin-fixed paraffin-embedded tissue sections from human prostate samples. Tissues sections were deparaffinized and submitted to immunohistochemistry procedures using the ImmPRESS Excel Amplified HRP Polymer Staining Kit (antirabbit IgG kit: MP7601; Vector Laboratories, Burlingame, CA, USA). Antigen retrieval was performed by heating the slides in an autoclave at 121 °C for 15 min in citric acid buffer (2 mM citric acid and 9 mM trisodium citrate dehydrate, pH 6.0). Antigen detection was undertaken according to the manufacturer’s instructions. The antigen–antibody complex was visualized with a 3,3-diaminobenzidine (DAB) solution (1 mM 3,3′-diaminobenzidine, 50 mM Tris HCl buffer, pH 7.6, and 0.006% H_2_O_2_).

The ratio of positive cells/total cells (0, none; 1, < 1/100; 2, 1/100 to 1/10; 3, 1/10 to 1/3; 4, 1/3 to 2/3; 5, > 2/3) and intensity of signals (0, none; 1, weak; 2, moderate; 3, strong) of positively stained cells were evaluated in every slide. The total scores (the sum of both scores) were used as immunoreactivity (IR score; 0, 2–8) in this study. The RFC2 IR was considered “high” when the IR score was 4 or higher. A score of 4 was considered the cut-off point, as the median value of the sum score was 3, and the mean score was 3.1. The optimal cut-off value in the receiver operating characteristic curve analysis for predicting cancer-specific survival was IR 4 in Pca patients. Two observers (M.O. and Y.Y.) evaluated the slides, and a third observer (T.F.) estimated the scores of the slides in case of disagreement between the 2 observers. The following commercially available antibody was used: rabbit anti-RFC2 (ab251796, Abcam).

### Statistical analyses

All statistical analyses were performed in Microsoft Excel (Microsoft) or GraphPad Prism software version 9.0 (GraphPad Software, Inc.). For RNA-seq and qRT-PCR analysis using clinical samples, Mann–Whitney test was performed to determine *P*-value. Two-sided Student’s *t*-test was performed to determine the statistical significance between two groups. When more than two samples were compared, a one-way analysis of variance (ANOVA) with post-doc Dunnett's multiple comparisons test was performed. Cancer-specific survival curves were obtained by the Kaplan–Meier method and verified by the log-rank (Mantel–Cox) test. Other statistical tests are described in figure legends. All data are expressed as the mean and SD, unless otherwise indicated. *P*-values < 0.05 were considered to be statistically significant.

### Supplementary Information

Supplementary Information.

## Data Availability

The remaining datasets generated during and/or analyzed during the current study are available from the corresponding author on reasonable request.
