# Supplementary material for: Identification of DNA damage response-related genes as biomarkers for castration-resistant prostate cancer
Source: Sci Rep. 2023 Nov 10;13:19602. doi: 10.1038/s41598-023-46651-6 (PMC10638319; doi:10.1038/s41598-023-46651-6)
Supplement: Supplementary file 1 — Supplementary Information. [file 41598_2023_46651_MOESM1_ESM.pdf]

Supplementary Information

Identification of DNA damage response-related genes as biomarkers for castration-resistant prostate cancer

Short title: DNA damage response-related genes in CRPC

Masashi Oshima, Ken-ichi Takayama, Yuta Yamada, Naoki Kimura, Haruki Kume, Tetsuya Fujimura, Satoshi Inoue

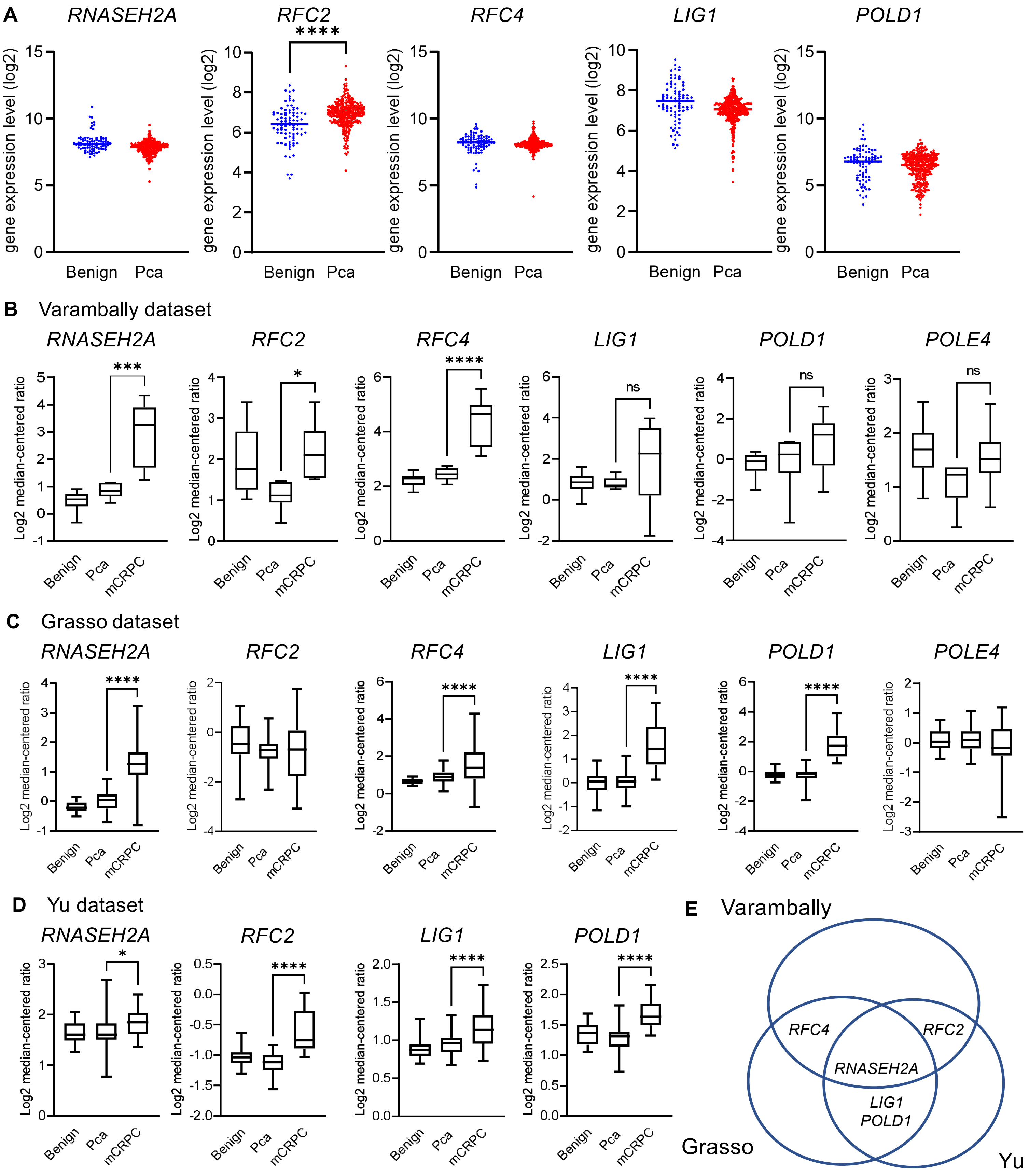

Supplementary Figure S1. Evaluation of DDR-related gene expression in normal and Pca tissues via public database

(A) The tissue-wide gene expression pattern of DDR-related genes across paired tissue samples was analyzed by using Gene Expression database of Normal and Tumor tissue 2 (GENT2) database. Red dots indicate cancer samples and blue indicates benign samples. ****$P < 0.0001$, ns: not significant.

(B-D) *RNASEH2A, RFC2, RFC4, LIG1, POLD1* and *POLE4* expression levels in Pca tissues were analyzed using a public database ((B): GSE3325, (C): GSE35988, (D): GSE6919). The P value was determined by Mann-Whitney U test. Bars, SD. *$P < 0.05$, **$P < 0.01$, ***$P < 0.001$, ****$P < 0.0001$. mCRPC: metastatic castration-resistant prostate cancer.

(E) Venn diagram showing the overlap of DDR-related genes that were found to be upregulated in CRPCs in the three datasets of public database (GSE3325, GSE35988, GS6919) are shown.

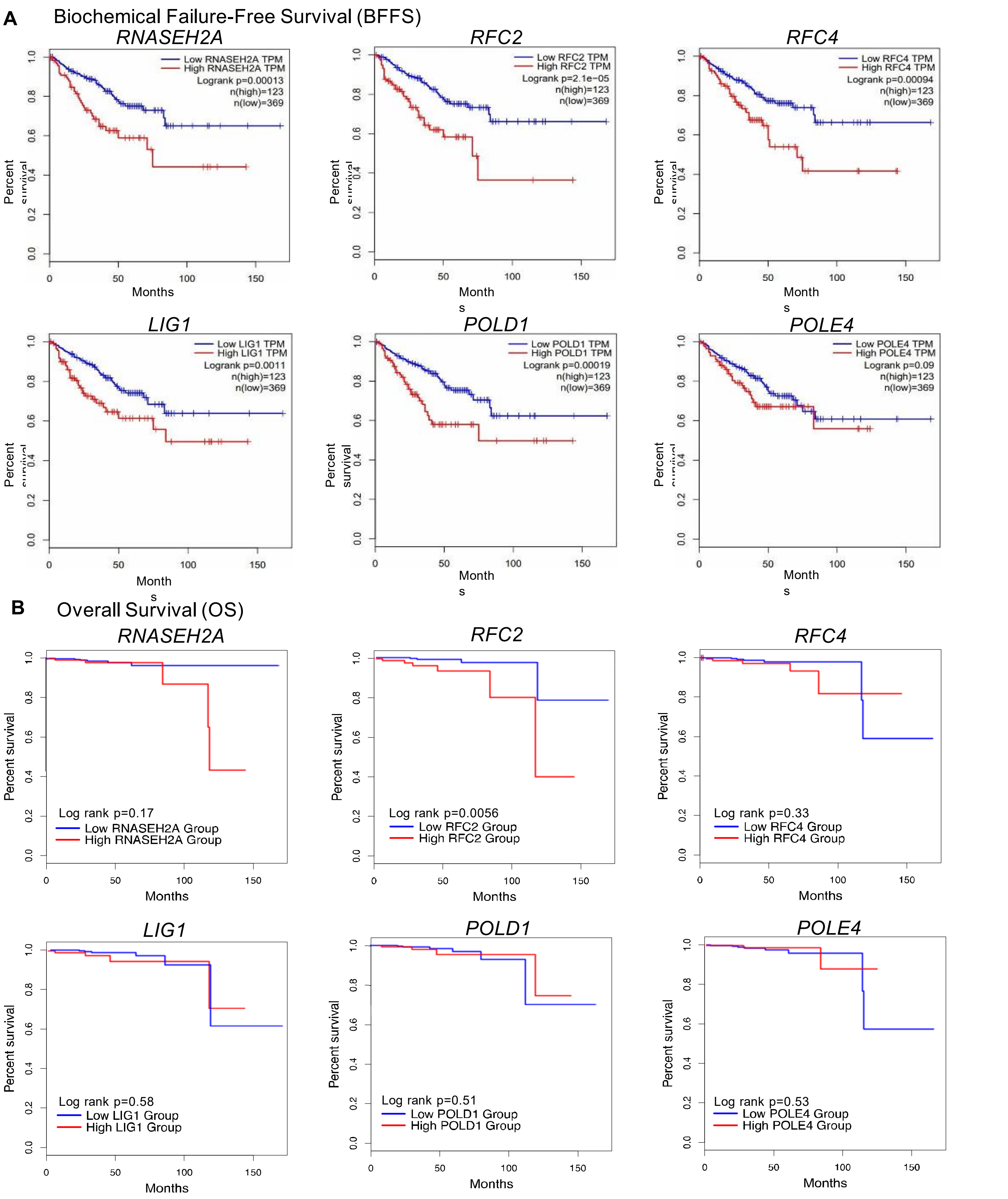

Supplementary Figure S2. Prognostic analysis of DDR-related genes expression in prostate cancer

(A) Biochemical Failure-Free Survival (BFFS) and (B) Overall survival (OS) analysis of DDR-related genes in prostate cancer. GEPIA2 generates Kaplan Meier curves comparing the groups with different expression levels of DDR-related genes (*RNASEH2A, RFC2, RFC4, LIG1, POLD1,* and *POLE4*) in TCGA Pca samples. Blue line: low expression groups (Bottom 75% cutoff); red line: high expression groups (Top 25% cutoff). Log-rank test was used to determine the *P*-values. The Cox proportional hazard ratio and the 95% confidence interval information were also included in the survival plots. $P < 0.05$ was considered to be statistically significant.

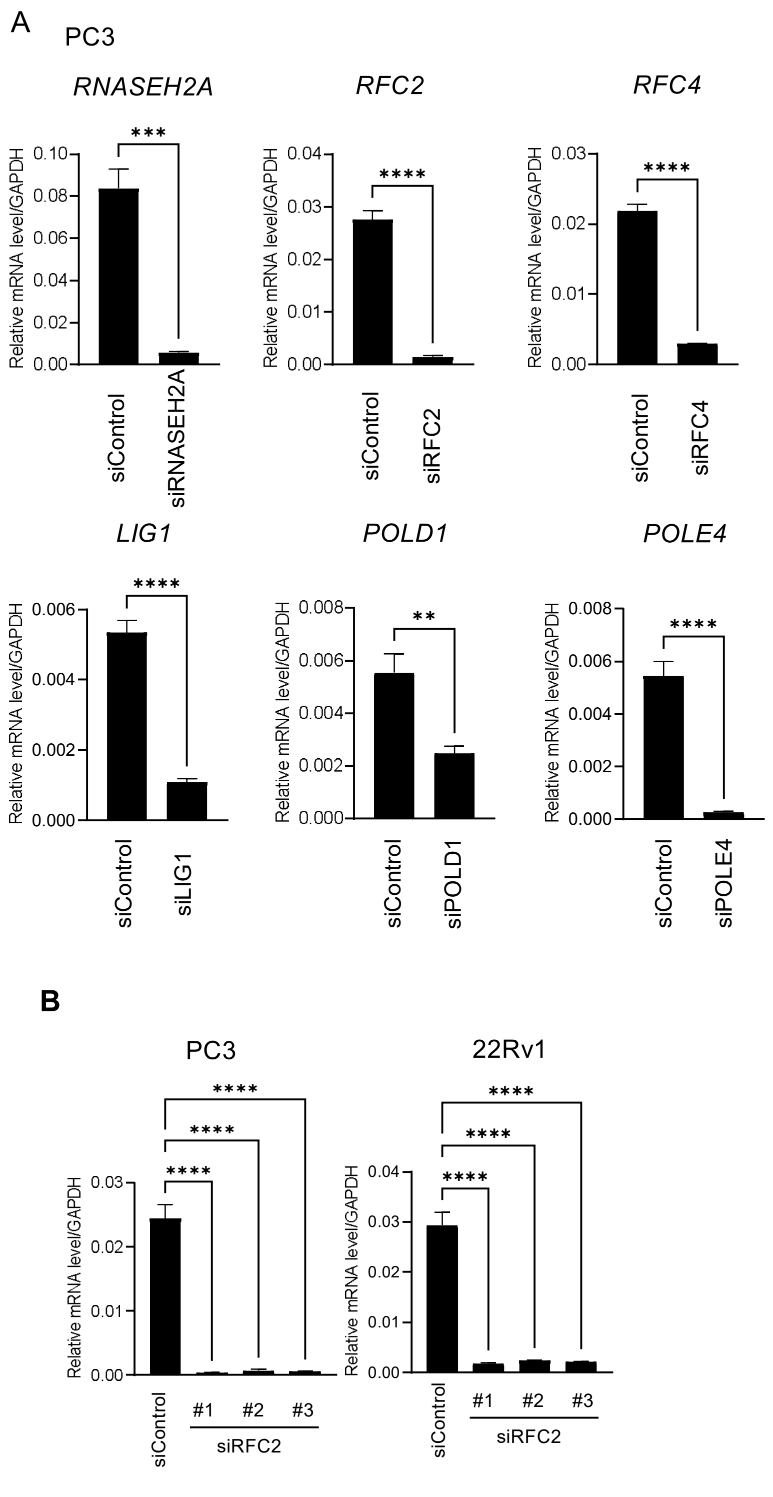

Supplementary Figure S3. Validation of the efficiency of gene expression suppression by each siRNA

(A) Validation of siRNA targeting DDR-related genes (*RNASEH2A, RFC2, RFC4, LIG1, POLD1, POLE4*). PC3 cell were transfected with 5 nM siControl or each siRNA targeting DDR-related genes for 48 h. We then measured mRNA expression level of each DDR-related gene by qRT-PCR. The results are presented as mean and SD (N = 3). $^{**}P < 0.01$, $^{***}P < 0.001$, $^{****}P < 0.0001$, compared with siControl.

(B) Validation of siRNA targeting RFC2. PC3 and 22Rv1 cells were transfected with 5 nM siControl or siRFC2 (#1, #2, and #3) for 48 h. We then measured mRNA expression level of RFC2 by qRT-PCR. The results are presented as mean and SD (N = 3). $^{****}P < 0.0001$, compared with siControl.

A

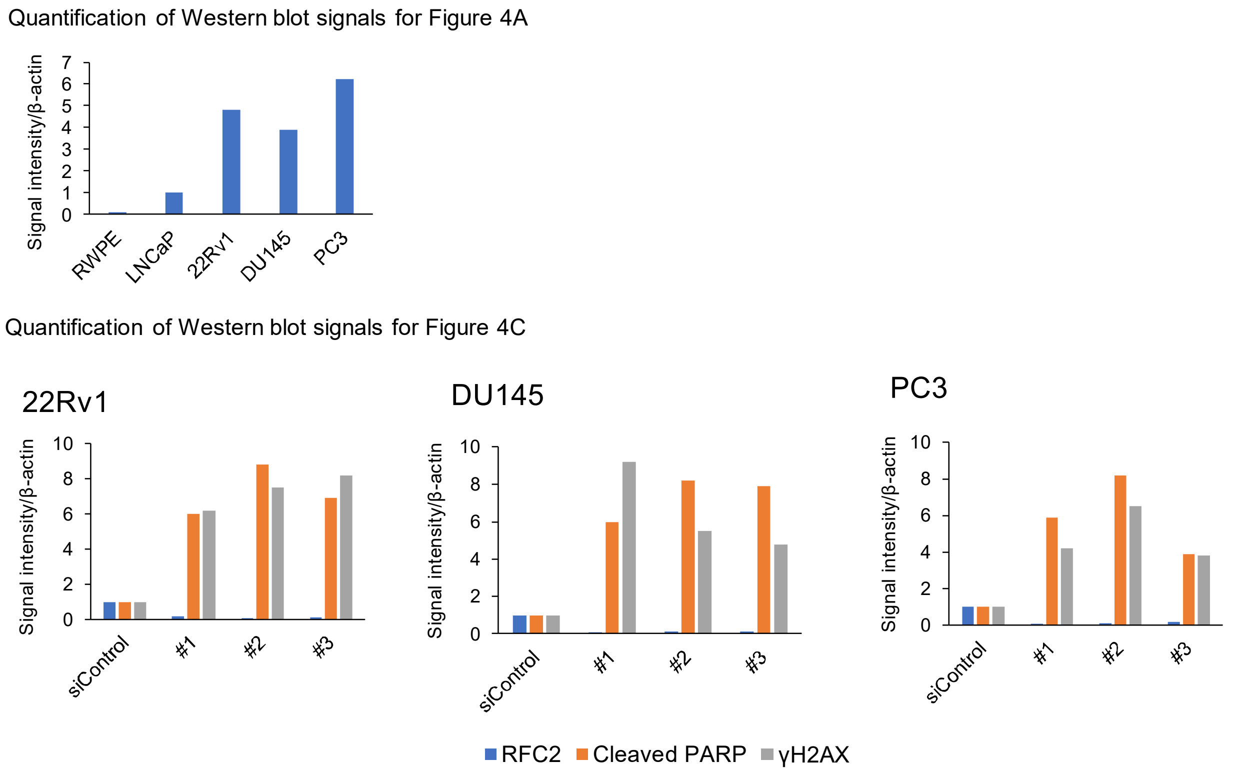

B

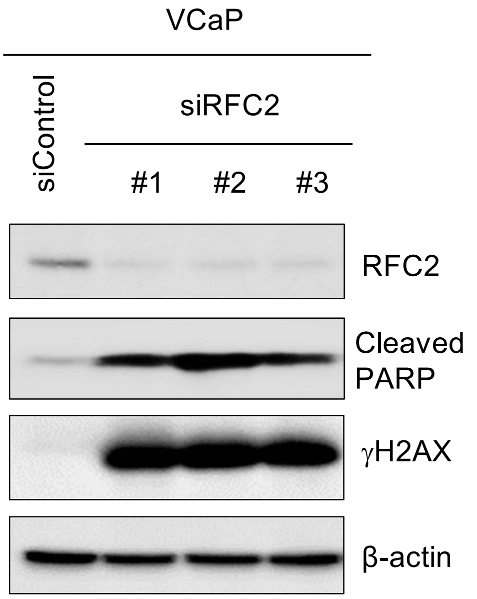

C

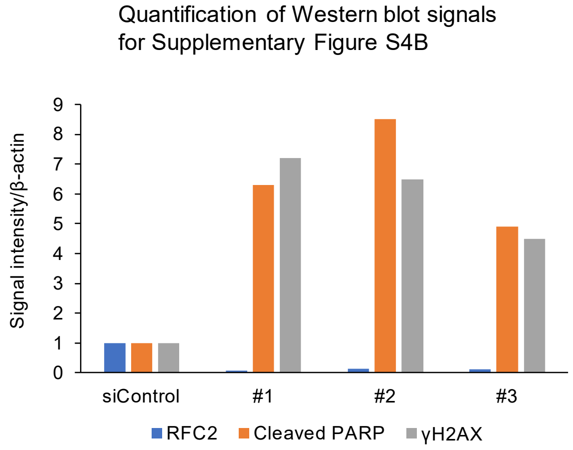

Supplementary Figure S4. Knockdown of RFC2 results in accumulation of DNA damage, increased apoptosis in CRPC cells

(A) Quantification of protein expressions in western blot analysis in Figure 4A and 4C. β-actin was used for normalization of protein expressions.

(B) Knockdown of RFC2 increases DNA damage and apoptosis in Pca cell lines. VCaP cells were transfected with 5 nM siControl or siRFC2 (#1, #2, and #3) for 72 h. Western blot analysis for RFC2, cleaved PARP and γH2AX was carried out and β-actin was used as a loading control.

(C) Quantification of protein expressions in western blot analysis in VCaP cells. β-actin was used for normalization of protein expressions.

Fig.3B

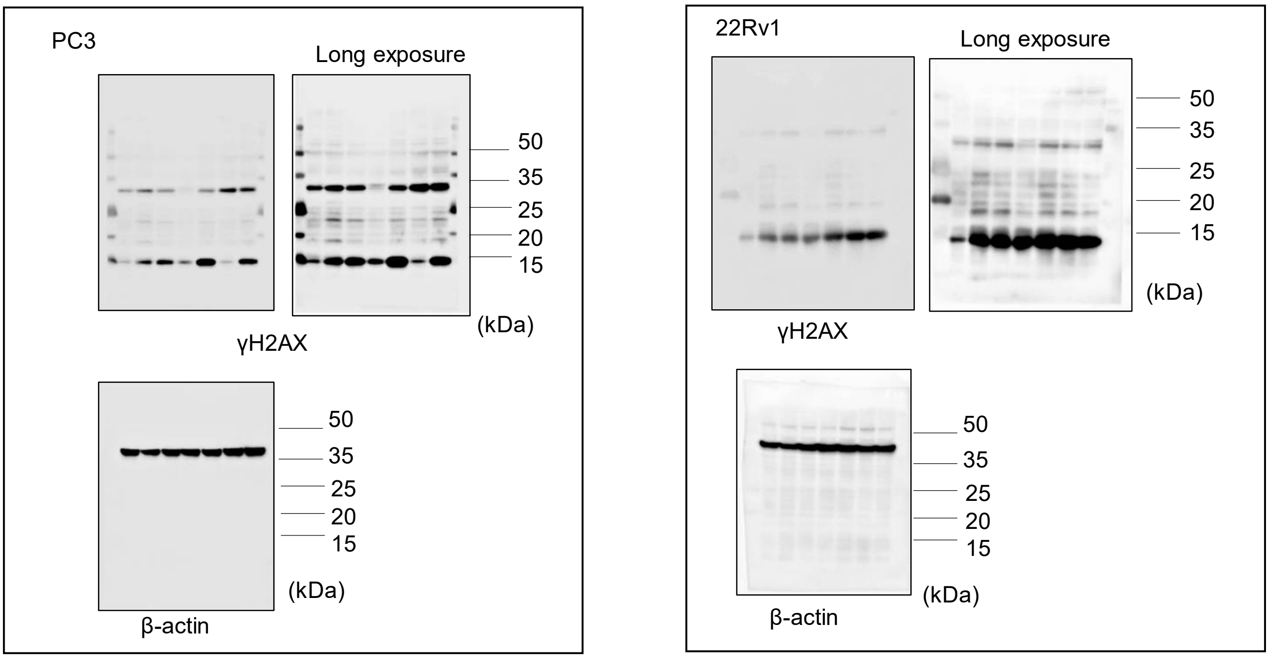

Fig. 4A

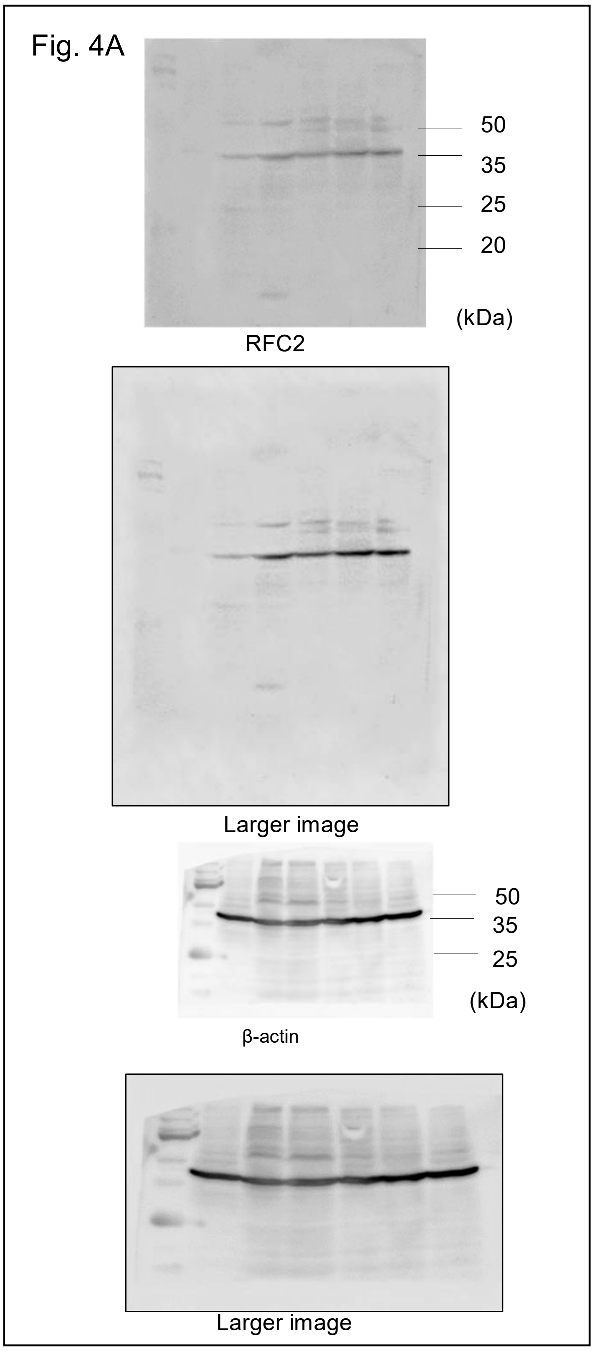

Fig. 4B

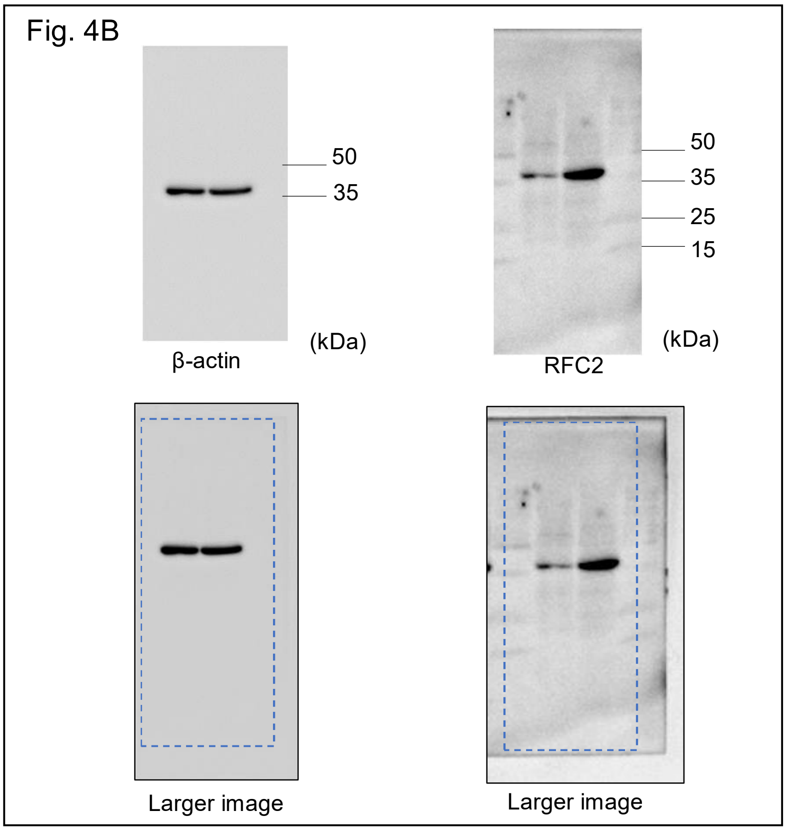

Supplementary Figure S5. Full scan images of Western blotting.

Fig. 4D

22Rv1

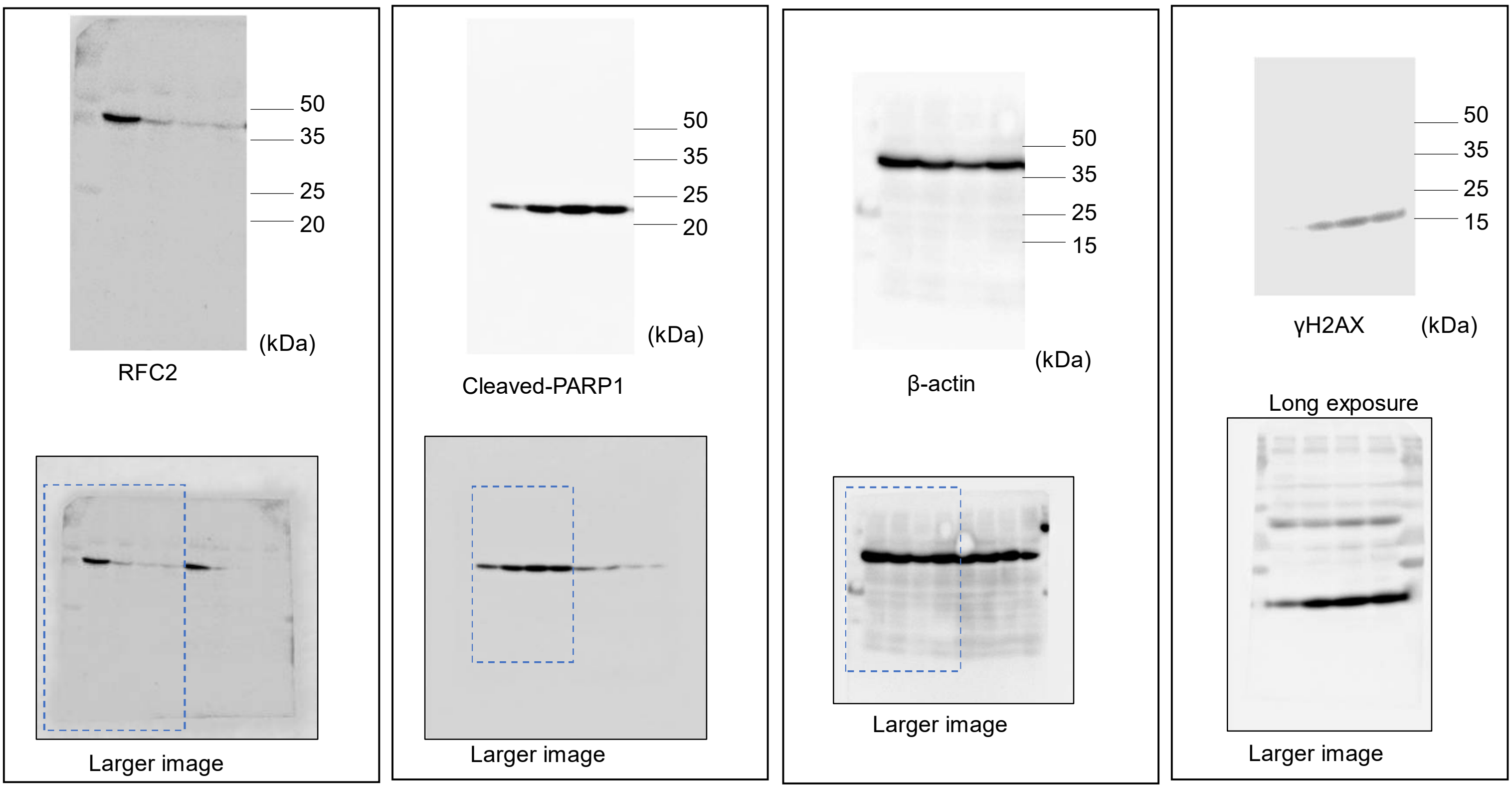

DU145

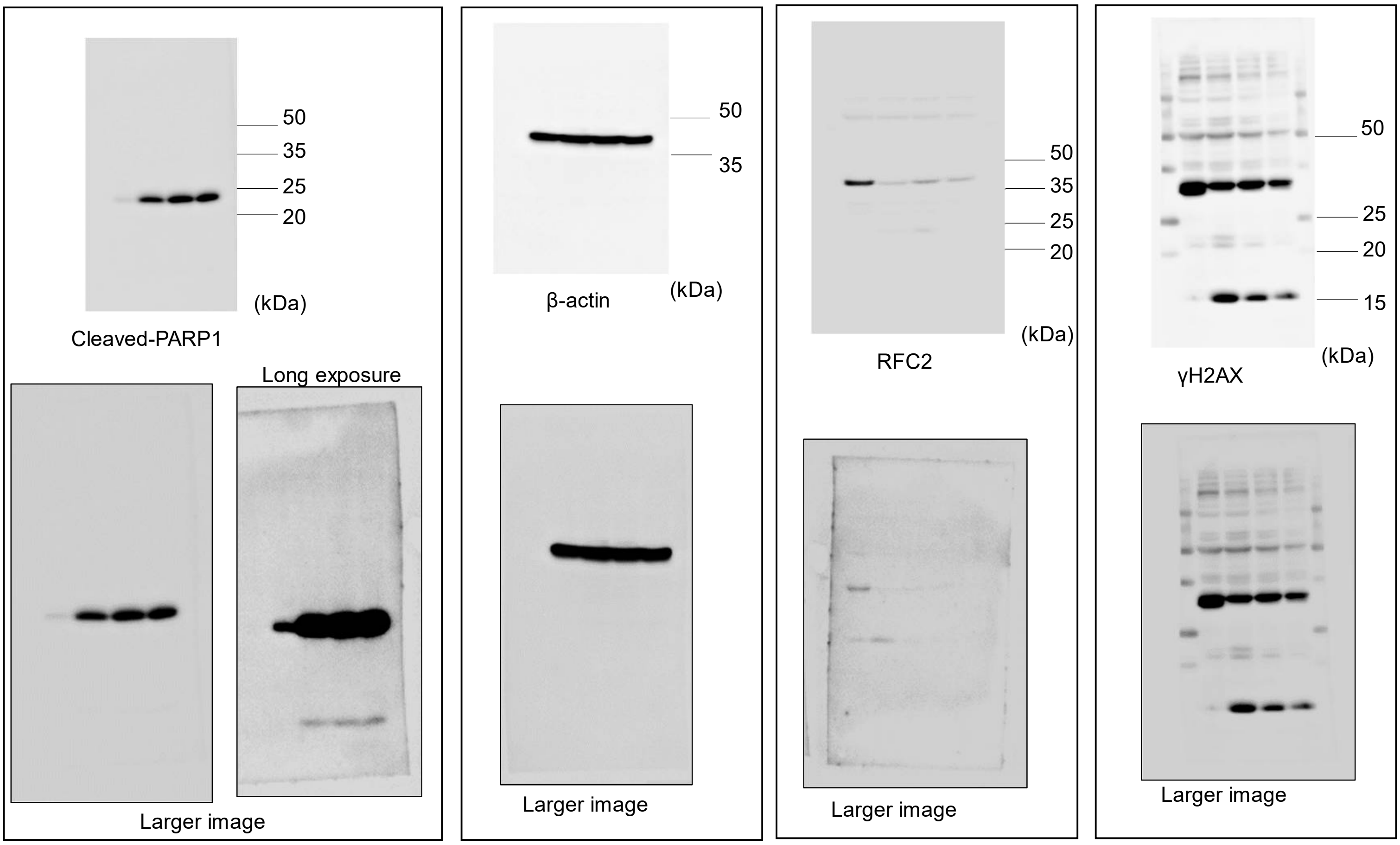

Supplementary Figure S5. Full scan images of Western blotting (continued).

PC3

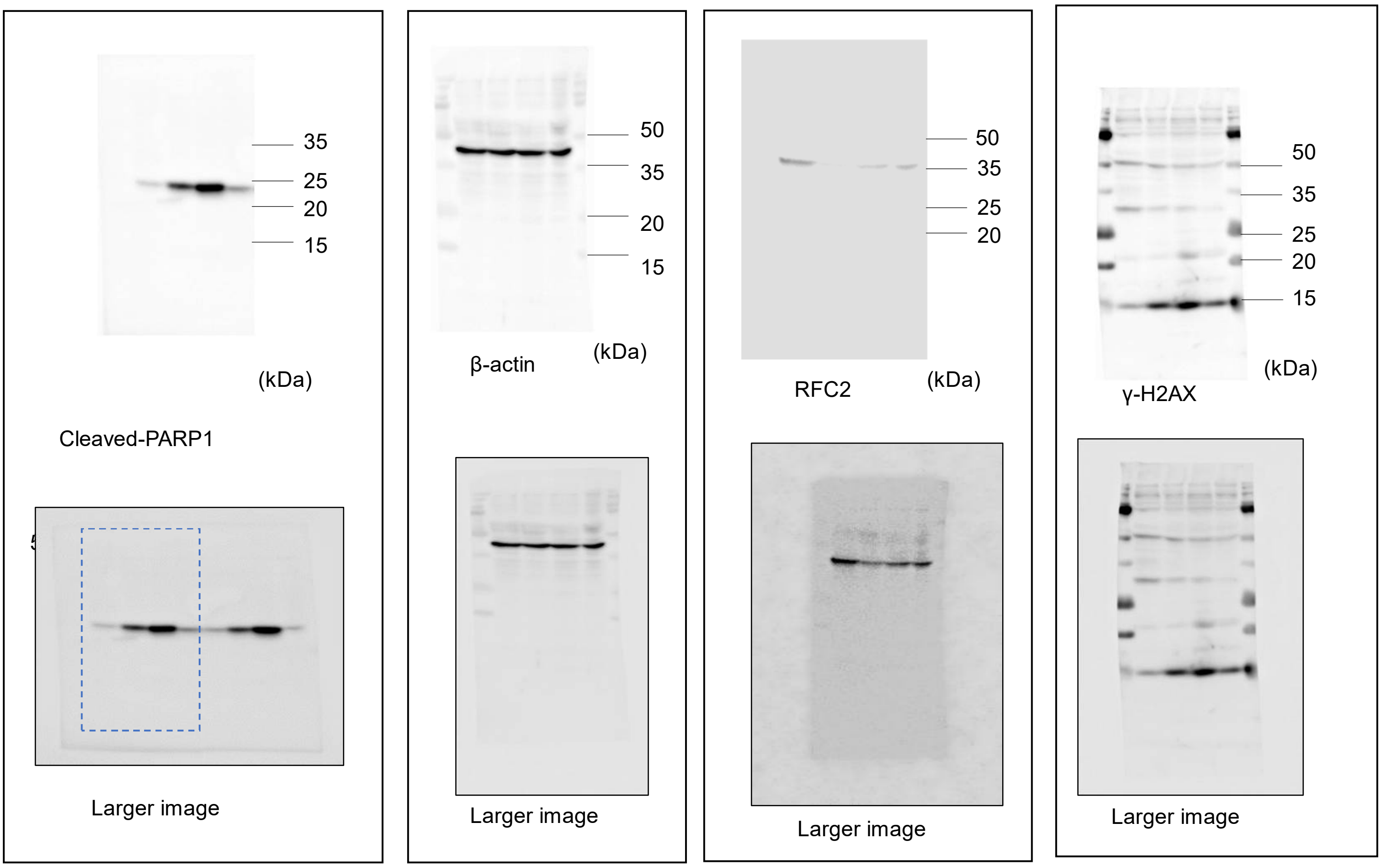

Supplementary Fig. 4B

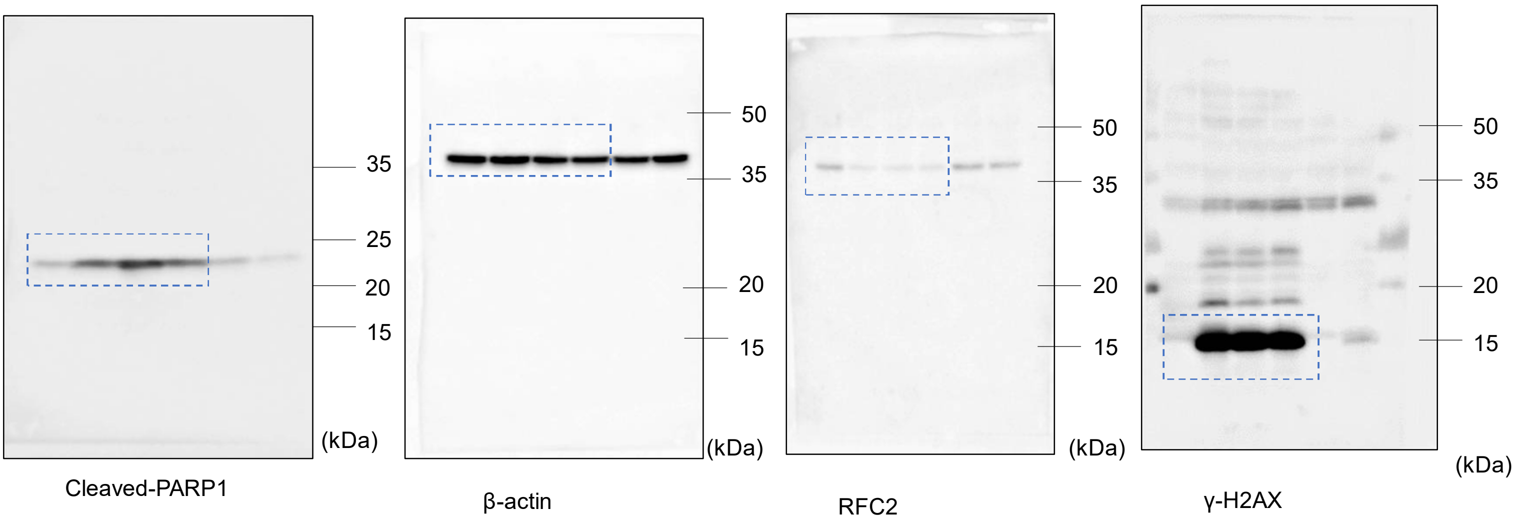

Supplementary Figure S5. Full scan images of Western blotting (continued).

Supplementary Table 1. Univariate and multivariate analyses for progression-free survival in prostate cancer patients in TCGA data.

	Univariate		Multivariate	
Parameters	HR (95% CI)	*P*-value	HR (95% CI)	*P*-value
Pathological Gleason score (pGS) (pGS ≥ 8 vs pGS < 8)	5.18 (3.17-8.72)	<0.0001	4.49 (2.46-8.45)	<0.0001
T stage (T ≥ 3b vs < 3b)	2.79 (1.74-4.47)	<0.0001	1.33 (0.73-2.42)	0.3547
N stage (N1 vs N0)	1.80 (1.01-3.15)	0.0462	0.84 (0.43-1.60)	0.5953
RFC2 mRNA expression (Top 50% vs Bottom 50%)	2.12 (1.33-3.44)	0.0015	1.47 (0.86-2.53)	0.1619

Univariate and multivariate analyses were evaluated using Cox's proportional hazard model. *P*-value < 0.05 was considered to be statistically significant. CI: confidence interval, HR: hazards ratio, PSA: prostate specific antigen, RFC2: replication factor C subunit 2.
